# Organic Farming: Biodiversity Impacts Can Depend on Dispersal Characteristics and Landscape Context

**DOI:** 10.1371/journal.pone.0135921

**Published:** 2015-08-26

**Authors:** Ruth E. Feber, Paul J. Johnson, James R. Bell, Dan E. Chamberlain, Leslie G. Firbank, Robert J. Fuller, Will Manley, Fiona Mathews, Lisa R. Norton, Martin Townsend, David W. Macdonald

**Affiliations:** 1 Wildlife Conservation Research Unit, The Recanati-Kaplan Centre, Department of Zoology, University of Oxford, Tubney, United Kingdom; 2 British Trust for Ornithology, Thetford, Norfolk, United Kingdom; 3 Centre for Ecology and Hydrology, Lancaster Environment Centre, Lancashire, United Kingdom; 4 Royal Agricultural University, Cirencester, Gloucestershire, United Kingdom; Towson University, UNITED STATES

## Abstract

Organic farming, a low intensity system, may offer benefits for a range of taxa, but what affects the extent of those benefits is imperfectly understood. We explored the effects of organic farming and landscape on the activity density and species density of spiders and carabid beetles, using a large sample of paired organic and conventional farms in the UK. Spider activity density and species density were influenced by both farming system and surrounding landscape. Hunting spiders, which tend to have lower dispersal capabilities, had higher activity density, and more species were captured, on organic compared to conventional farms. There was also evidence for an interaction, as the farming system effect was particularly marked in the cropped area before harvest and was more pronounced in complex landscapes (those with little arable land). There was no evidence for any effect of farming system or landscape on web-building spiders (which include the linyphiids, many of which have high dispersal capabilities). For carabid beetles, the farming system effects were inconsistent. Before harvest, higher activity densities were observed in the crops on organic farms compared with conventional farms. After harvest, no difference was detected in the cropped area, but more carabids were captured on conventional compared to organic boundaries. Carabids were more species-dense in complex landscapes, and farming system did not affect this. There was little evidence that non-cropped habitat differences explained the farming system effects for either spiders or carabid beetles. For spiders, the farming system effects in the cropped area were probably largely attributable to differences in crop management; reduced inputs of pesticides (herbicides and insecticides) and fertilisers are possible influences, and there was some evidence for an effect of non-crop plant species richness on hunting spider activity density. The benefits of organic farming may be greatest for taxa with lower dispersal abilities generally. The evidence for interactions among landscape and farming system in their effects on spiders highlights the importance of developing strategies for managing farmland at the landscape-scale for most effective conservation of biodiversity.

## Introduction

Farmland biodiversity in Europe has been declining for some time, with a particularly steep trend in the second half of the twentieth century. The consensus is that agricultural intensification is responsible; the evidence for this is extensive. For example, countries with higher wheat yields have more threatened or recently extinct arable weed species [1] and plant species richness is negatively related to nitrogen input at a country-level scale [2]. Bird population declines and range contractions have been greatest in countries with the highest cereal production [3], [4]. With ever greater pressures to produce food, compounded by globalisation of markets, biofuels, climate change [5] and agricultural specialisation [6], these trends are likely to continue. Recent incentives and agri-environment schemes introduced to address this issue have halted and even reversed declines in some groups [7] and, while in some parts of Europe agriculture is not intensified and may not have contributed to substantial biodiversity loss [8], in general, farmland biodiversity continues to decline in Europe [9], [10], amid continued concerns over the potentially harmful effects of pesticides [11]. Furthermore, there is concern that the new agricultural policy of the European Union will not sufficiently address this issue—indeed, it may be less beneficial for biodiversity [12]. There is therefore a continuing need to refine initiatives which may benefit farmland biodiversity.

Intensification has brought increased use of pesticides and fertilisers, changes in cropping patterns (such as a shift from spring-sown to winter-sown crops and a reduction in the use of traditional crop rotations), land drainage, pasture improvement, and the loss of non-cropped habitats [13], [14]. Disentangling which aspects of intensification are responsible for biodiversity loss is challenging. Chamberlain et al. [15] implicated land use changes as a factor explaining bird declines, with a lag between the changes and their impact on bird populations. Geiger et al. [16] explored 13 components of intensification and found that insecticides and fungicides have had the most consistent negative effects. Neonicotinoid pesticides have attracted particular attention [17]. Conducting field trials of appropriate statistical power in this area is difficult, yet it is difficult to infer the implications for wildlife populations from laboratory studies [18]. A recent study of farmland in the Netherlands [11] demonstrated that cascading trophic effects on non-target species (birds) may be more severe than previously suspected. Benton et al. [19] reviewed the evidence for the impact of removal of non-cropped habitats from farmland. They concluded that loss of heterogeneity at multiple spatial scales was likely to have been influential for a range of taxa.

Organic farming is a form of low intensity farming which has attracted attention for its generally wildlife-friendly practices. It takes a systems approach to management and relies on the use of crop rotations, green manure, compost, and biological pest control. Some types of fertilizers and pesticides are used but they are strictly limited compared to conventional farming. Cropping patterns differ markedly: there is a greater emphasis on spring sowing of cereals, whereas autumn-sowing in conventional farming has greatly increased over the past 60 years, and organic crop rotations typically include a fertility-building ley [20]. In addition, organic farms in the UK have greater areas of non-cropped habitat, more grassland, and a higher density of boundary and hedges (resulting from smaller field sizes). The hedges on organic farms tend to be higher and wider, have fewer gaps, and have more trees and woody shrubs compared to those on conventional farms [20]. Some practices on organic farms may not be especially wildlife-friendly, for example, heavy mechanical weed control (see [21]).

Organic farming can benefit a range of taxa. Reviews describe a tendency for organic farms to have higher species richness and abundance of plants, invertebrates, and birds [22–25]. There are, however, two important caveats to be considered before concluding that organic farming, or some elements of it, can help reverse the negative impact of farming on biodiversity (also noting that organic yields are often lower than conventional yields with implications for the balance between farmland productivity and wildlife conservation (e.g. [26])). Firstly, the farming effects vary greatly among taxa. In Fuller et al.’s study [27], we found that the largest and most consistent effects were for plants and the smallest and least consistent were for carabid (ground) beetles. Schneider et al. [28] observed consistent effects for plants and bees, with less consistent effects for earthworms and spiders. A review concerning Central and Northern Europe found that predatory groups were more likely to be ‘losers’ than ‘winners’ of organic farming where diversity was concerned, and carabid beetles were one of the groups affected negatively [24].

The second major complication for promoting organic farming, or elements of it, to encourage biodiversity, is that the effect may vary across the landscape. Most studies for which data are available on this have demonstrated that the benefits are greater in simple, less heterogeneous landscapes (e.g. [29] for arable weeds; [30] for butterflies). Schneider et al.’s study [28] found highest gains (for plants, bees, earthworms and spiders) in intensive arable fields. For policy, it would be useful to know whether landscape targeting of lower intensity farming is worthwhile. It would also be useful to know whether conventional farmers can deploy elements of organic farms (such as the amount or quality of uncropped habitats) to achieve biodiversity gains, or whether features of the organic system that are not easily transferrable to a conventional system, such as restricted pesticide use, are more likely to be responsible.

Carabids and spiders are promising groups for illuminating the relationship between landscape, farm management and biodiversity. Both groups are surface-active and abundant in arable fields, where they contribute to pest control (e.g. [31]). Furthermore, their numbers and diversity are likely to indicate those of their prey (most carabid beetles and all spider species are predatory), especially small invertebrates including mites, Collembola, and aphids. Both local farming practices and landscape scale intensification of agriculture are expected to interact to affect spider and ground beetle communities (for example, see hypotheses in [32]). Local habitat factors, such as vegetation, microclimate and management, may affect the abundance and richness of species of more limited dispersal capabilities [33], and the quality and structure of the surrounding landscape has been shown to be important for species with higher dispersal abilities [34]. Linyphiidae (spiders which generally have higher dispersal potential, often dispersing aerially by ballooning) were found to be more independent from landscape characteristics in the closer surroundings than the cursorial Lycosidae [35]. A number of studies have shown the importance for spiders and carabids of non-crop habitat features: for example, uncropped field margins have been shown to be particularly important both for the activity density of lycosids and for the species richness and composition of linyphiids [36]. Weibull et al. [37] found the species richness of taxonomic groups including spiders and carabid beetles generally to increase with landscape heterogeneity on a farm scale. Brooks et al. [38] observed links between carabids and their trophic links that were consistent at large scales, enhancing their value as an indicator group, and Jonason et al. [39] showed that carabid species richness was influenced more by the wider landscape than by local factors. Geiger et al. [16] recorded more species where a higher proportion of the landscape was managed under agri-environment schemes. In a large scale study across Europe [40], carabids were more abundant (but not more species rich) in simple landscapes, while farming system had no detectable effect on either abundance or species richness. In contrast, a study of twelve pairs of farms in Germany found that the species richness (but not abundance) of ground-dwelling spiders in crop fields decreased with landscape simplicity irrespective of farming system; more spider species were recorded where the surrounding landscape had a higher proportionate area of non-cropped habitats [41]. Batary et al. [42] similarly found hunting spiders were more abundant when intensively managed farmland was scarce in the surrounding landscape, an effect which was stronger on conventional farms.

In this paper we pursue the findings of Fuller et al. [27], where we reported the effects of organic farming across a range of taxonomic groups. We found that there were postive effects on spider activity density and species density, while the effects of farming system for carabids were inconsistent. Here, we analyse data from the same large sample of paired organic and conventional farms in the UK that we used in Fuller et al [27]. We ask, first, how the observed effects of organic farming on spiders and carabids were related to the landscape context of the farms in the study and, second, if any effects of farming system on spiders and carabids could be associated with non-crop habitat farming system differences. To explore further the effects of local and landscape-scale impacts of organic farming on spiders we categorised species in one of two basic guild categories: hunting or web-building. This classification has been used in other studies (e.g. [43]. Batary et al. [43] describe this as a well-founded classification, since spiders using these different behaviours to catch their prey also differ in the resources they use and the way in which they use them (see [44] for a review of the classification of spiders of agricultural fields into guilds). As noted in [43], Oberg et al. [36] showed that landscape variables are important determinants of species composition of lycosid and linyphiid spiders, which represent two of the most important groups of hunting and web-building spiders respectively, and which differ in their dispersal potential (lycosids with lower dispersal abilities than linyphiids). Although the classification is relatively coarse (Uetz et al. [44] describe the challenges associated with assigning spiders to guilds), it is meaningful in a study investigating the local and landscape impacts of organic farming on spiders.

## Methods

### Site selection and sampling methods

The basic approach was a large-scale comparison, during the period 2000–2003, of organic and conventional farms paired on the basis of proximity, crop type and cropping season [27]. Permission was given by all farmers/landowners to conduct the study on their land. Organic farms of at least 30 ha with contiguous organic fields containing arable land were identified from the databases of the Soil Association and Organic Farmers and Growers. Data were collected from 60 pairs of farms; 80% of pair members were within 10 km of one another (median distance = 6.4 km) but pair members were non-contiguous. All conventional farms in our sample used either herbicides, insecticides or both. One organic and one conventional winter wheat field were randomly selected (‘target fields’) in each of the 60 farm pairs. Plants and invertebrates were sampled on these 60 pairs of fields.

Invertebrates were sampled over two years, using pitfall traps (trapping effort was the same in each target field in both years). Although experiments have shown that pitfall trap catches can be affected by a number of factors such as differing activity rates and habitat structure [45], pitfall trapping is nonetheless a valuable and widely used method for sampling spiders (e.g.[46]) and carabid beetles (e.g. [47]). A grid of 18 pitfall traps was set in each target field, comprising nine within the cropped area and nine within the uncropped boundary (hereafter referred to as ‘crop’ and ‘boundary’ samples, respectively). Traps were set for one week before emptying. Paired target fields were always sampled at the same time. Because of seasonal variation in animal activity and trapping efficiency, samples were collected both before and after harvest (in 2002, pre-harvest sampling extended between 13^th^ May and 23^rd^ July, and post-harvest sampling between 2^nd^ September and 16^th^ October; in 2003, pre-harvest sampling extended between 23^rd^ May and 9^th^ August and post-harvest sampling between 1^st^ September and 17^th^ October). There were therefore four trapping periods in total, over the two years of the study, each trapping period lasting one week (approximately 168 hours). Spiders and carabid beetles were identified to species, using keys [48] and [49] respectively. Target fields were sampled for plant species richness in the same two years. Boundary plots recorded plant species richness of non-crop species in plots extending 1 m from the centre of the uncropped field boundary and 10 m parallel to the boundary. Plant species richness of non-crop plants within the cropped area were recorded from five 0.5 x 0.5 m quadrats placed in the crop on 12 transects per field. The height and width of the hedgerow at 10 locations next to the pitfall traps were also recorded, as was the width of the uncropped field boundary.

### Analysis

We calculated the mean number of individuals in the nine replicate traps in the boundary and the mean number of individuals in the nine replicate traps in the cropped area, separately for pre- and post-harvest, within the target field. We calculated the mean species richness in the same way. In common with Fuller at al. [27], we refer to these metrics from here on as activity density and species density respectively (following Gotelli and Colwell [50]). Spider species were assigned to one of two broad guilds. Hunting spiders, which include the wolf spiders (Lycosidae), generally disperse on the ground by walking, termed cursorial. Web builders, which include the linyphiids, frequently disperse long distances by ballooning [51]. Although this is a coarse classification (as discussed earlier), this separation allowed us to explore patterns in the data which might suggest different local and landscape impacts of organic farming on groups with potential differences in dispersal characteristics.

We compared a series of candidate mixed models using the model selection procedure of [52], [53]. Relative model performance was assessed using the Akaike Information Criterion (AIC), adjusted for sample size (AIC_C_). Competing models were ranked using Akaike weights. These are interpreted as the probability that the model in question is the ‘best’ model of the dataset, among the series of models considered. The process accounts for model uncertainty, and the resulting set estimates are ‘unconditional’; they do not depend on any single model (unless one model is clearly dominant). Our selection of candidate models was based on competing and biologically plausible hypotheses.

First we identified the best model from candidate models including only farming system and two aspects of landscape structure. The percentage of arable land around each farm was used as a metric of landscape complexity (heterogeneity), as for some previous studies (e.g. [40]). Models also included the percentage of woodland as a candidate predictor, as there was an *a priori* expectation that woodland may provide refuge habitat for mobile species [33]. We used the percentage of these land use categories at two different spatial scales (1 km^2^ and 9 km^2^) around the focal farm. These data were derived from the Land Cover Map 2000 integrated with CS2000 field survey data 2000 [54–56]. The % cover of arable land and woodland in the 1 km^2^ containing the target field (target square) and in the 3 km x 3 km with the target square at its centre—the 9 km^2^ scale—were used as predictors. Table 1 provides descriptive statistics for all the response and predictor variables used in the analyses.

**Table 1:** Descriptive statistics for response variables (spiders and carabids), and predictor variables (landscape and field level) for conventional and organic farms. N = 60 farm pairs.

	Conventional						Organic					
	mean	SE	min	max	LCI	UCI	mean	SE	min	max	LCI	UCI
**Spiders**												
**Before harvest**												
Boundary hunters												
Activity density	4.81	0.70	0.00	22.70	3.39	6.22	6.38	1.08	0.20	36.60	4.23	8.55
Species density	1.84	0.16	0.00	4.67	1.52	2.16	2.05	0.18	0.22	5.44	1.69	2.40
Cropped area hunters												
Activity density	3.64	0.69	0.20	37.10	2.25	5.03	6.80	0.70	0.30	24.20	5.39	8.21
Species density	1.50	0.12	0.22	4.56	1.25	1.74	2.39	0.15	0.29	4.78	2.09	2.69
Boundary web-builders												
Activity density	5.66	0.63	0.30	18.30	4.39	6.93	5.37	0.64	0.10	21.80	4.09	6.65
Species density	2.27	0.15	0.22	4.56	1.98	2.57	2.28	0.17	0.11	6.22	1.93	2.62
Cropped area web-builders												
Activity density	9.18	0.97	0.70	31.00	7.23	11.12	8.34	0.84	0.10	25.70	6.65	10.02
Species density	2.89	0.15	0.56	4.89	2.58	3.20	2.72	0.16	0.13	5.22	2.41	3.03
**After harvest**												
Boundary hunters												
Activity density	0.76	0.13	0.00	3.60	0.51	1.02	1.13	0.15	0.00	3.10	0.83	1.44
Species density	0.46	0.06	0.00	1.43	0.33	0.58	0.66	0.07	0.00	1.63	0.51	0.81
Cropped area hunters												
Activity density	1.89	0.34	0.10	8.90	1.21	2.58	2.18	0.38	0.10	11.90	1.42	2.95
Species density	0.77	0.08	0.11	2.00	0.61	0.93	0.99	0.11	0.11	3.00	0.78	1.20
Boundary web-builders												
Activity density	0.85	0.08	0.20	2.60	0.68	1.02	1.11	0.19	0.00	6.20	0.72	1.50
Species density	0.71	0.06	0.22	1.56	0.59	0.83	0.67	0.07	0.00	1.78	0.53	0.82
Cropped area web-builders												
Activity density	1.12	0.29	0.10	10.60	0.53	1.71	0.82	0.13	0.00	3.40	0.55	1.09
Species density	0.70	0.08	0.11	2.11	0.53	0.86	0.64	0.09	0.00	2.22	0.47	0.82
**Carabids**												
**Before harvest**												
Boundary												
Activity density	15.92	1.59	0.67	60.78	12.73	19.11	17.30	1.89	1.11	80.38	13.52	21.09
Species density	4.41	0.20	0.67	9.33	4.00	4.81	4.09	0.22	0.89	8.63	3.66	4.52
Cropped area												
Activity density	30.89	2.67	3.00	98.10	25.05	35.73	38.50	3.31	5.70	108.00	31.87	45.12
Species density	5.47	0.27	2.00	10.78	4.93	6.02	5.74	0.18	2.89	8.78	5.37	6.10
**Carabids**												
**After harvest**												
Boundary												
Activity density	4.92	0.50	0.67	13.11	3.90	5.94	4.48	0.66	0.00	15.67	3.14	5.82
Species density	2.61	0.19	0.67	5.78	2.22	3.00	2.18	0.20	0.00	4.89	1.78	2.58
Cropped area												
Activity density	10.39	1.06	1.00	28.78	8.24	12.54	10.22	1.40	1.25	43.22	7.37	13.06
Species density	3.44	0.20	0.78	6.22	3.03	3.85	3.21	0.21	0.75	6.22	2.79	3.64
**Predictor variables**												
**Landscape (% area)**												
Arable, 1 km2	49.46	2.66	14.92	87.77	44.13	54.79	41.2	2.3	5.12	84.77	36.58	45.81
Arable 9 km2	45.68	2.22	16.23	82.71	41.25	50.12	42.74	2.01	13.09	81.73	38.7	46.77
Woodland, 1km2	7.18	0.58	1.45	24.82	6.02	8.35	8.33	0.62	2.85	26.02	7.1	9.56
Woodland, 9 km2	7.93	0.47	2.83	22.8	6.98	8.88	8.55	0.44	2.49	19.92	7.66	9.44
**Field**												
Hedgerow bulk*	3.63	0.55	0	20.15	2.54	4.73	3.52	0.4	0	11.8	2.72	4.32
Margin width (m)	2.54	0.22	0.61	8.06	2.1	2.97	2.47	0.17	0.78	6	2.13	2.82
Plant species richness (cropped area)	5.32	0.23	1.67	9	4.87	5.78	13.01	0.63	0	22.83	11.75	14.27
Plant species richness (boundary)	13.4	0.58	5	26	12.24	14.56	15.69	0.67	7	30	14.36	17.03

* sqrt(mean width x mean height

With 4 landscape variables and 1 system variable, there are 5 possible main effects models with one predictor, 8 possible models with two predictors, and 10 possible models with three predictors. The screening out of models that contain the same landscape attribute at different scales removes zero, 8 and 6 models respectively from that set. The remaining 17 main effects models generate a further 16 models with interaction terms between system and landscape. Including the null model, the final model set therefore comprises 34 models.

Model averaging using the ‘natural’ averaging method [52] was used to generate parameter estimates for system and landscape effects. Continuous predictors were standardised and all models were forced to include calendar year as a blocking effect, and farm pair identity as a categoric random factor. The abundance response was log-transformed, and the species density response square root transformed. Where there was evidence for interaction between system and land use (the confidence interval for the parameter estimate not including zero), we fitted models for organic and conventional farms separately.

Where we identified farming system effects, as we reported in Fuller et al. [27], or revealed here via interaction with landscape (evidence for a farming system effect restricted to some landscape types), we compared the model including farming system (and landscape where appropriate) with models including the non-crop habitat variables described above, and also the plant species richness measures. For cropped area responses, the habitat predictor used was plant species richness, following previous work (e.g. [41]). Plants provide both habitats and food resources for invertebrates (e.g. [38]). For field boundary responses, the habitat predictors were boundary width and hedge bulk (square root of the product of the mean width and mean height of the hedgerow). Interaction terms with farming system were included to allow for the possibility that the habitat effect differed with farming system. To establish evidence that any habitat variable explains a farming system difference, we would expect to see both that that predictor variable differed between farming system, and that it was influential *within* farming system. The R software was used for analyses [57], with normal errors mixed models fitted using the lme4 package [58]. Model selection and model averaging methods were applied with the MuMin package [59].

## Results

From the winter cereal sampling, we identified 131 species of spider (n = 29,377 individuals) and 107 species of carabid beetle (n = 62,162 individuals). More spiders and carabids were captured within the crop compared with the uncropped field boundary, particularly so for carabids. This was true for both years. For example, in 2002, an average of 12.9 carabids (SE = 1.4) and 12.1 (SE = 1.10) spiders per trap were captured in the field boundary (before harvest) compared with 34.0 (SE = 3.3) carabids and 14.8 (SE = 0.80) spiders in the cropped area. In 2003, the mean capture rates in the boundary were 20.5 (SE = 1.90) and 10.4 (SE = 0.92) respectively for carabids and spiders, compared with 35.3 (SE = 2.68) and 14.1 (SE = 1.22) in the cropped area. Trapping rates were between approximately 5–10 times higher before harvest compared with after harvest for both groups. The post harvest trapping rates in the cropped area in 2002 were 8.54 (SE = 0.74) for carabids and 3.21 (SE = 0.41) for spiders, with similar rates observed in 2003.

### Farming system effect varies with landscape type

Both farming system and landscape affected spiders, but the patterns for hunting and web-building spiders were different. For hunting spiders, farming system and landscape (extent of arable land) were both statistically significant predictors. Extent of arable land measured at either 1 km^2^ or 9 km^2^ were equally good predictors (one of these appeared in many of the high ranked models, Table 2).

**Table 2:** Model selection outputs for responses with significant system effect or system-landscape interaction. Top 3 models in descending order of Akaike weight given for each such response variable. Predictor variables: 1- arabkm1z (arable in 1 km x 1 km square around farm), 2- arabkm3z (arable in 3 km x 3 km square around farm), 3- calyear (calendar year, 2002,2003), 4- system (organic, conventional), 5- woodkm1z (woodland in 1 km x 1 km square around farm), 6- woodkm3z (woodland in 3 km x 3 km square around farm). Interaction terms: 7- arabkm1z:system, 8- arabkm3z:system, 9- system:woodkm1z, 10- system:woodkm3z. In the presence of system-landscape interaction, landscape models are given for each system separately. Model averaged parameter estimates given in S1 Table.

	Deviance	AICc	Delta	Weight
**SPIDER MODELS**				
**Hunting spiders. Before harvest activity density in cropped area.**				
2+3+4+8	206.13	221.18	0.00	0.33
2+3+4+5+8	204.99	222.36	1.18	0.18
2+3+4+6+8	205.67	223.04	1.86	0.13
**Organic farms only:**				
3	24.29	119.58	0.00	0.32
3+5	24.03	121.28	1.70	0.14
3+6	24.05	121.34	1.76	0.13
**Conventional farms only:**				
2+3	20.22	111.46	0.00	0.42
1+3	20.87	113.27	1.81	0.17
2+3+5	20.14	113.63	2.17	0.14
**Hunting spiders. Before harvest species density in cropped area.**				
2+3+4+6+8	62.29	79.66	0.00	0.22
2+3+4+8	65.43	80.48	0.83	0.14
2+3+4+5+8	63.18	80.56	0.90	0.14
**Organic farms only:**				
2+3	6.75	48.94	0.00	0.22
1+3	6.81	49.43	0.48	0.18
2+3+5	6.64	50.38	1.44	0.11
**Conventional farms only:**				
2+3+6	5.79	42.61	0.00	0.19
1+3+6	5.83	43.01	0.41	0.15
1+3	6.09	43.08	0.47	0.15
**Hunting spiders. Before harvest activity density in field boundary.**				
2+3+4+5+8	247.74	265.12	0.00	0.11
1+3+4+5+7	247.75	265.13	0.01	0.11
2+3+4+8	250.23	265.28	0.17	0.10
**Organic farms only:**				
2+3	32.71	138.87	0.00	0.34
2+3+5	31.71	139.5	0.63	0.25
2+3+6	31.96	139.95	1.08	0.20
**Conventional farms only:**				
3+5	30.93	135.67	0.00	0.23
3	32.72	136.58	0.90	0.15
3+4	31.54	136.79	1.12	0.13
**Hunting spiders. Before harvest species density in field boundary.**				
2+3	130.43	140.99	0.00	0.12
2+3+5	128.41	141.19	0.21	0.11
2+3+6	128.48	141.26	0.28	0.11
**Organic farms only:**				
2+3	10.25	72.73	0.00	0.46
2+3+5	10.09	74.23	1.50	0.22
2+3+6	10.19	74.79	2.05	0.16
**Conventional farms only:**				
3+5	11.07	77.1	0.00	0.32
1+3+6	10.88	78.53	1.44	0.16
3	11.94	79.1	2.00	0.12
**Hunting spiders. After harvest activity density in field boundary.**				
1+3+4+6+7	58.83	76.98	0.00	0.34
1+3+4+6+7+10	58.45	79.18	2.20	0.11
1+3+4+5+7	61.24	79.39	2.41	0.10
**Organic farms only:**				
1+3+4	5.06	43.11	0.00	0.27
1+3	5.47	43.38	0.26	0.24
1+3+5	5.22	44.29	1.18	0.15
**Conventional farms only:**				
1+3+6	4.59	39.38	0.00	0.30
1+3	5.00	39.97	0.59	0.22
3	5.39	40.31	0.93	0.19
**Hunting spiders. After harvest species density in field boundary.**				
1+3+4+6+7+10	22.85	43.58	0.00	0.24
1+3+4+6+7	25.74	43.89	0.31	0.21
3+4+6	31.3	44.52	0.95	0.15
**Organic farms only:**				
1+3	3.22	23.3	0.00	0.21
3+6	3.27	23.83	0.54	0.16
3	3.54	24.33	1.04	0.12
**Conventional farms only:**				
1+3+6	2.95	22.55	0.00	0.74
3+6	3.51	26.5	3.95	0.10
3	3.91	28.14	5.59	0.05
**CARABID BEETLE MODELS**				
**Before harvest activity density in cropped area.**				
2+3+4	234.62	247.41	0.00	0.12
3+4	237.19	247.75	0.34	0.10
2+3+4+8	233.48	248.54	1.13	0.07
**Before harvest species density field boundary.**				
2+3+4	92.76	105.53	0.00	0.19
2+3	95.48	106.03	0.50	0.15
1+3+4	94.73	107.5	1.97	0.07
**After harvest activity density in field boundary.**				
1+3+4	132.16	145.37	0.00	0.11
3+4	134.76	145.62	0.24	0.10
1+3+4+6	130.28	145.93	0.56	0.08
**After harvest species density in field boundary.**				
3+4	73.96	84.82	0.00	0.19
1+3+4	73.31	86.52	1.71	0.08
3+4+6	73.61	86.82	2.01	0.07

**Fig 1:**
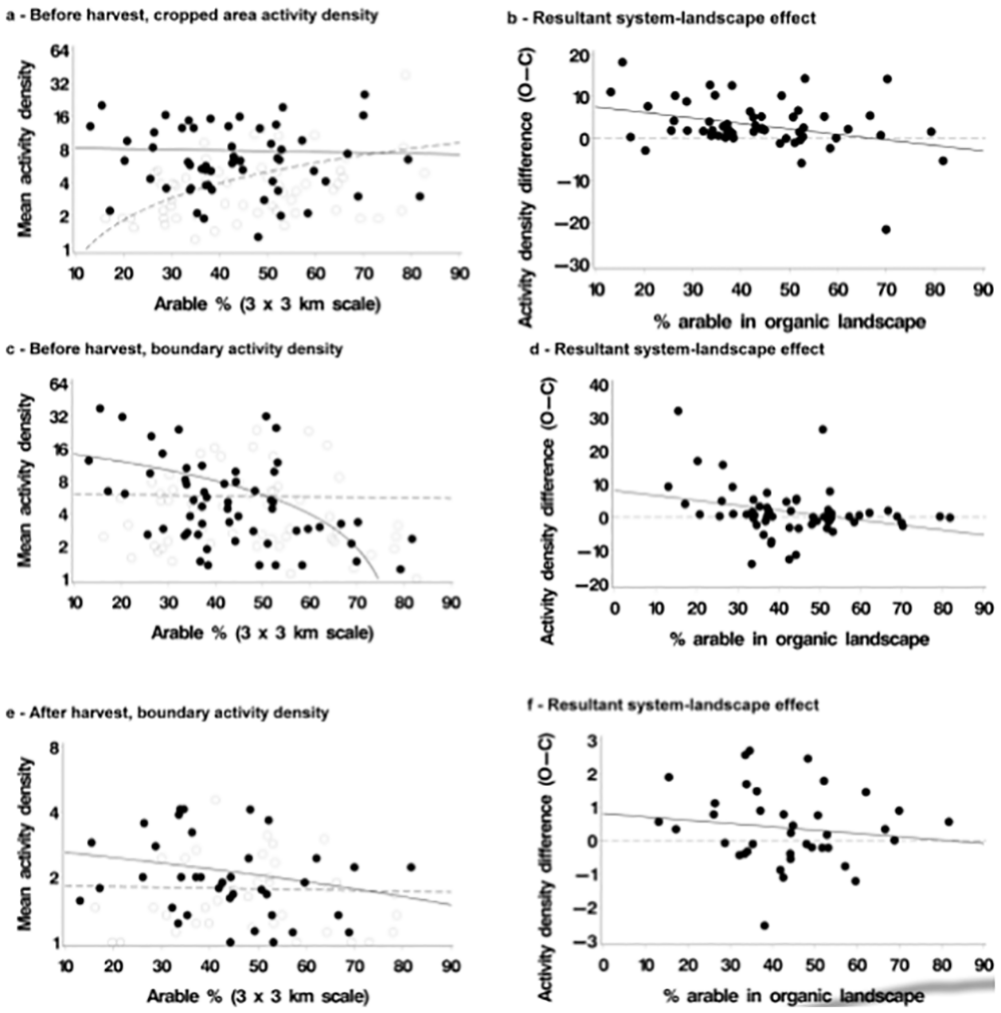
Effects of farming system and landscape (% arable) on activity density of hunting spiders where system effect is significant. Organic: solid lines, filled points; conventional: dotted line, open points. Plots in left hand panel: lines in plots are linear regressions plotted on log scale by system, points are farms. Plots in right hand panel: lines in plots are linear regression (solid line) and reference line (dotted at system difference = zero), points are farm pairs. Right hand panel extracts system effect from left hand panel, O-C difference on arithmetic scale.

More hunting spiders were captured, and more species of hunting spiders captured, on organic compared to conventional farms; the farming system effect was particularly marked in the cropped area before harvest—our analysis suggested that an average of 77% more individuals and 36% more hunting spider species were captured on organic farms. In both crop and boundary samples before harvest, the effect of farming system depended on landscape type. The farming system difference (more hunting spiders were captured on organic compared to conventional farms) was more pronounced in less arable (more complex) landscapes (Fig 1a). In the cropped area, the effect was the result of there being more individuals and species captured across the continuum of arable extent, while there were fewer on conventional farms in arable-dominated landscapes.

This is clear if we plot the response difference for each pair of farms against the extent of arable land in the landscape (Fig 1b). Almost all differences were positive for hunting spiders in the cropped area, indicating a positive organic effect, regardless of landscape. For hunting spiders in boundary samples (both numbers of individuals captured and species density), the farming system effect was apparent only in complex landscapes (those comprised of less than approximately 40% arable land (Fig 1c); there was a clear system-landscape interaction); species density followed a pattern similar to that of activity density, for hunting spiders.

After harvest, no effects of farming system or landscape were detected on hunting spiders in the cropped area. In the boundary, however, there were more hunting spiders captured on organic farms and, again, this effect was clearer in complex landscapes (Fig 1e and 1f). In other words, there was a farming system effect (hunting spider activity density was higher on organic farms), while an interaction between farming system and landscape again was attributable to the farming system effect being larger in complex landscapes because of opposing trends with landscape complexity for organic and conventional farms—numbers declining with arable land extent on organic farms and increasing on conventional farms (Fig 1e), effects that were statistically significant or marginally so (*P* = 0.03 and *P* = 0.07 respectively, Table 3). The pattern was similar for species density (Table 3).

**Table 3:** Model averaged parameter estimates (back transformed) for farming system and landscape effects (extent of arable land in landscape). The responses were square root transformed mean species counts per trap (Species Density, SD), and log mean numbers per trap. Effect size = organic/conventional ratio (LCI = Lower Confidence Interval, UCI = Upper Confidence Interval). Confidence intervals not encompassing zero are emboldened.

	System parameter estimate	SE	System effect size	LCI	UCI	Landscape effect	SE
**Spiders before Harvest**							
Cropped area, hunter activity density	0.578	0.093	**1.78**	**1.49**	**2.14**	0.220§	0.081
Cropped area, hunter SD	0.310	0.050	**1.36**	**1.24**	**1.50**	0.067§	0.043
Boundary, hunter activity density	0.085	0.114	1.09	0.87	1.36	-0.116#	0.108
Boundary, hunter SD	0.039	0.066	1.04	0.91	1.18	-0.094#	0.057
Cropped area, web builder activity density	-0.066	0.096	0.94	0.78	1.13	none	
Cropped area, web builder SD	-0.043	0.056	0.96	0.86	1.07	none	
Boundary, web builder activity density	-0.023	0.088	0.98	0.82	1.16	none	
Boundary, web builder SD	-0.004	0.057	1.00	0.89	1.11	none	
§Main effects of arable positive (more spiders and species captured in more arable landscape), but with significant interaction indicating positive trend confined to conventional farms (Fig 1a). #Significant interaction, indicating negative trends with arable extent confined to organic farms (Fig 1e).							
**Carabids before Harvest**							
Cropped area, activity density	0.232	0.120	**1.26**	**1.00**	**1.60**	none	
Cropped area, SD	0.045	0.059	1.05	0.93	1.17	**-0.173§**	**0.035**
Boundary, activity density	0.002	0.126	1.00	0.78	1.28	None	
Boundary, SD	-0.012	0.007	0.99	0.97	1.00	-0.12§	0.004
§Fewer species captured in more arable landscapes							
**Spiders After Harvest**							
Cropped area, hunter activity density	0.118	0.143	1.13	0.85	1.49	none	
Cropped area, hunter SD	0.114	0.071	1.12	0.98	1.29	none	
Boundary, hunter activity density	0.165	0.080	**1.18**	**1.01**	**1.38**	0.113§	0.072
Boundary, hunter SD	0.156	0.066	**1.17**	**1.03**	**1.33**	-0.18§	0.075
Cropped area, web builder activity density	-0.120	0.075	0.89	0.77	1.03	**-0.167#**	**0.069**
Cropped area, web builder SD	-0.097	0.064	0.91	0.80	1.03	**-0.115#**	**0.047**
Boundary, web builder activity density	0.008	0.080	1.01	0.86	1.18	none	
Boundary, web builder SD	-0.095	0.053	0.91	0.82	1.01	**-0.092#**	**0.035**
§No overall effect of arable, but significant interaction indicating a positive trend on conventional farms. #Negative trends with increasing arable in landscape							
**Carabids after Harvest**							
Cropped area, activity density	-0.103	0.145	0.90	0.68	1.20	none	
Cropped area, species density	-0.066	0.072	0.94	0.81	1.08	none	
Boundary, activity density	-0.254	0.132	**0.78**	**0.60**	**1.00**	none	
Boundary, species density	-0.177	0.082	**0.84**	**0.71**	**0.98**	none	

**Fig 2:**
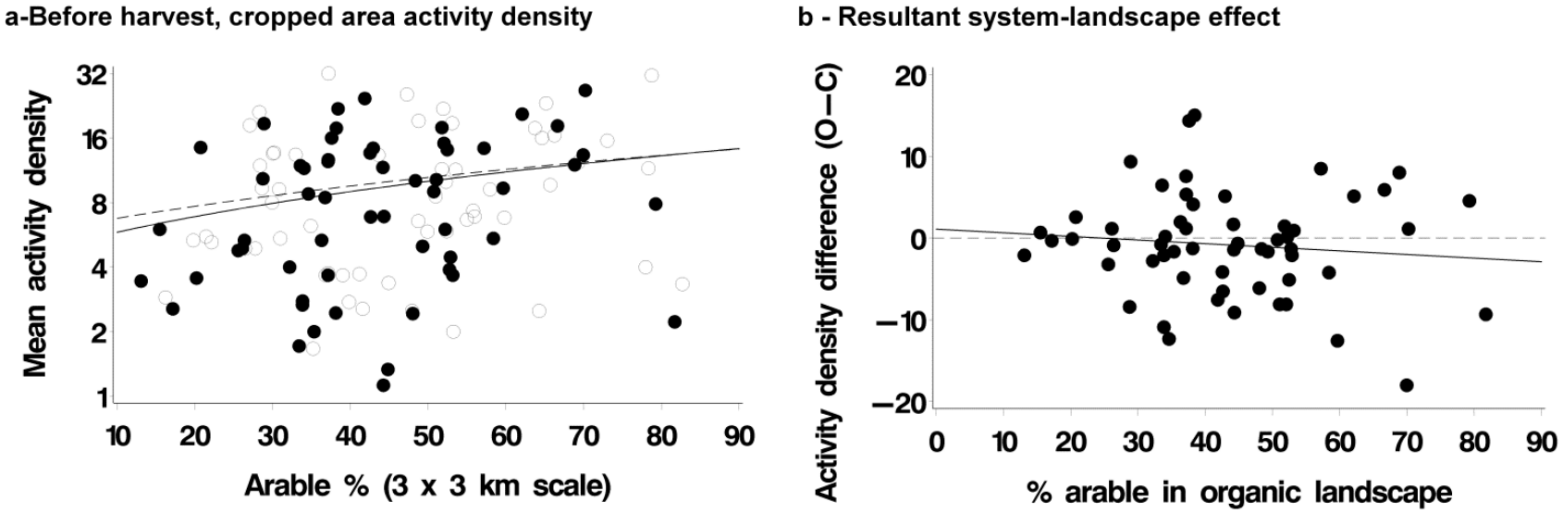
Effects of farming system and landscape on activity density of web-building spiders, no system effect, and one example plotted. Organic: solid lines, filled points; conventional: dotted line, open points. Plot in left hand panel: line in plot is linear regression plotted on log scale by system, points are farms. Plot in right hand panel: line in plot is linear regression (solid line) and reference line (dotted at system difference = zero), points are farm pairs. Right hand panel extracts system effect from left hand panel, O-C difference on arithmetic scale.

There was no evidence for any effect of farming system or landscape on web-building spiders in either the before or after harvest samples (Table 3). The pattern in the cropped area before harvest (Fig 2a and 2b) was therefore in marked contrast to that for the hunting spiders.

**Fig 3:**
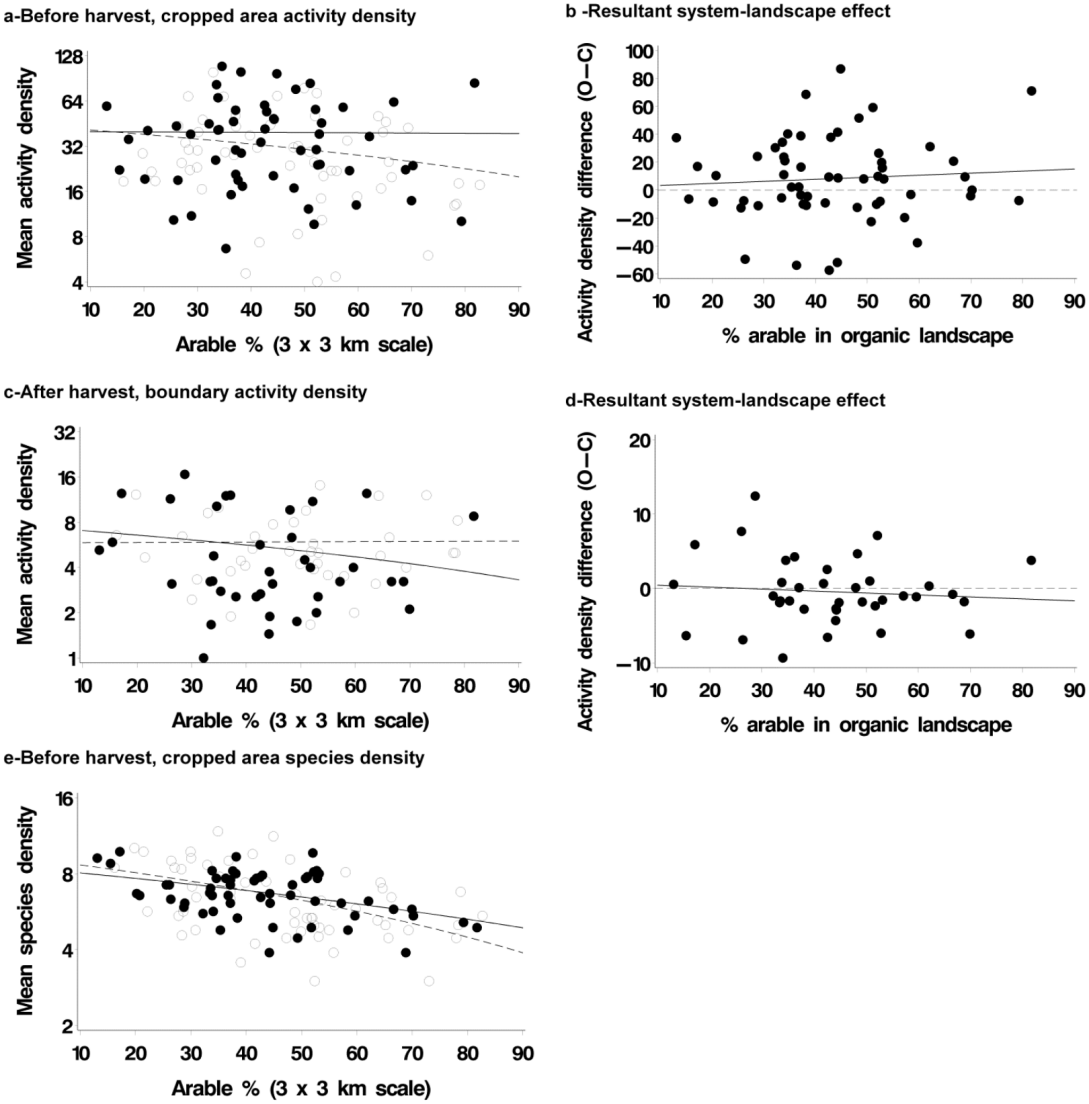
Effect of farming system and landscape on activity density of carabid beetles. Organic: solid lines, filled points; conventional: dotted line, open points. Plots in left hand panel: lines in plots are linear regressions plotted on log scale by system, points are farms. Plots in right hand panel: lines in plots are linear regression (solid line) and reference line (dotted at system difference = zero), points are farm pairs. Right hand panel extracts system effect from left hand panel.

The effect of farming system on carabid beetles was not consistent. Before harvest, more individuals were captured in the cropped area on organic farms, while the boundaries had similar numbers in organic and conventional farm systems (Table 3; Fig 3a and 3b). After harvest, fewer were captured on organic than conventional boundaries (Fig 3c and 3d; Table 3). There was no evidence for any interactions between landscape and farming system on carabid activity density; the observed farming system effects were therefore not specific to any landscape type. Carabids were less species-dense in more arable landscapes (Fig 3e); negative trends were observed for species density in both the cropped area and field boundaries (Table 3). There was little evidence that the amount of woodland in the landscape had any effect on either spiders or carabids (S1 Table).

### Does habitat explain the farming system effects?

The clearest farming system effect occurred for hunting spiders in the cropped area before harvest (Table 3). The only habitat variables we used which showed a clear field-level farming system difference were non-crop plant species richness measures (made pre-harvest). In the cropped area, the mean species richness recorded was 13.01 (SE = 0.63) on organic farms compared with 5.29 (SE = 0.23) on conventional farms (*F*_1,59_ = 156, *P* < 0.001). In the boundary, the farming system difference was less pronounced (organic mean 15.69 (0.67) species, conventional mean 13.27 (0.58), *F*_1,59_ = 3.95,*P* < 0.001). Field level hedge dimensions and boundary width were similar for both farming systems (Table 1).

**Fig 4:**
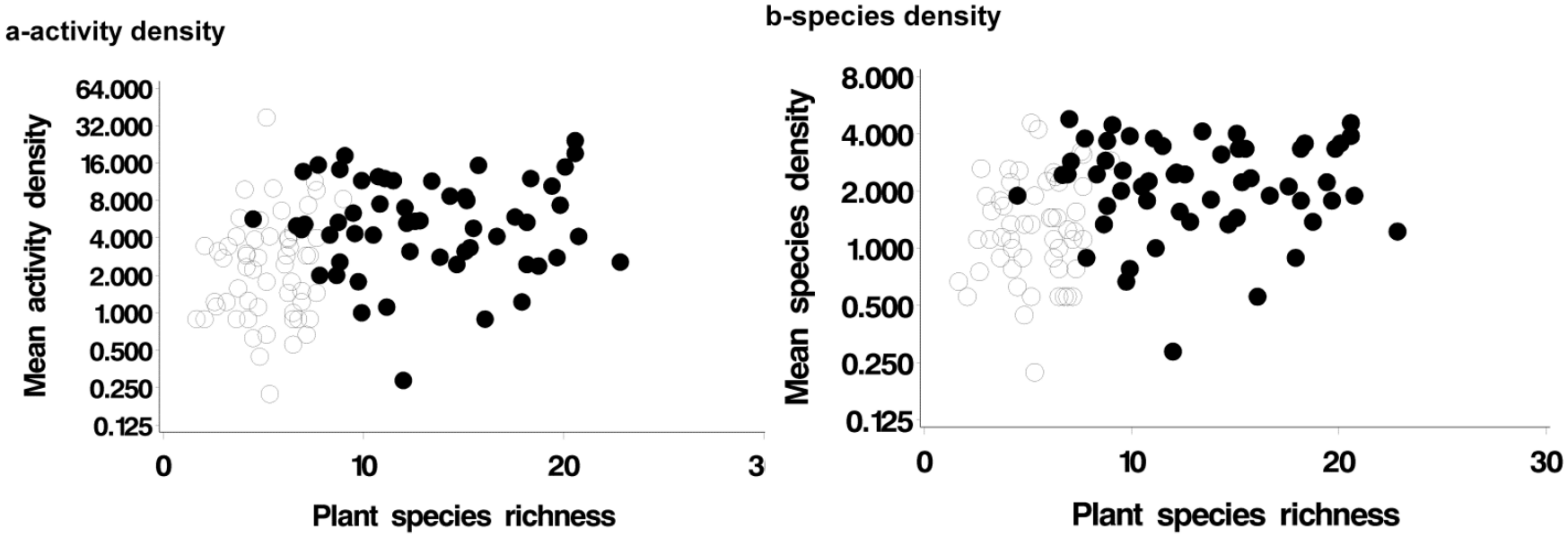
(a) Hunting spider activity density and field plant species richness (before harvest). Organic farms shown as solid points. Upward trend in conventional farms (open points, model averaged parameter estimate 0.10, SE = 0.05, P = 0.04), no trend among organic farms (model averaged parameter estimate 0.02, SE = 0.02, P = 0.29). (b) Hunting spider species density and field plant species richness (before harvest). Organic farms shown as solid points. Upward trend in conventional farms (open points, model averaged parameter estimate 0.05, SE = 0.03, P = 0.06), no trend among organic farms (model averaged parameter estimate 0.005, SE = 0.01, P = 0.66).

Models for hunting spiders in the cropped area before harvest, including plant species richness, were ranked higher than the ‘baseline’ system-arable model and the top models also included interaction between farming system and plant species richness (Table 2). In other words, there was some evidence that plant species richness within the crop was influential. Separate modelling for organic and conventional farms suggested plant species richness in the crop was positively correlated with hunting spider activity density on conventional farms, but not on organic (the parameter estimates were 0.10 SE = 0.049 and 0.02 (0.01) respectively). Farming system and plant species richness in the crop are highly correlated, i.e. organic fields tend to be weedy. The result is consistent with an upward trend in hunting spider species density on conventional farms where plant species richness is low, while no trend occurs over the range observed on organic farms (Fig 4a). A similar pattern was observed for hunting spider species density in the cropped area (Fig 4b). The plant species richness difference does not explain all the farming system effect, as the overall farming system effect remains influential in models which include plant species richness.

We explored how other farming system effects (Table 3), some varying across landscape types, were related to the plant species richness predictors (Table 1). For carabid activity density in cropped fields before harvest (which was higher on organic farms), there was an interaction between plant species richness and farming system. To explain the plant species richness interaction we looked at each system separately. There was a possible negative effect of plant species richness on organic farms (parameter estimate = -0.04, SE = 0.01, P = 0.06), and no effect on conventional farms (parameter estimate = 0.01, SE = 0.06, P = 0.86)). The field plant species richness difference is not, therefore, by itself, a compelling explanation for the farming system effect on carabid activity density. For other farming system effects, no links with plant species richness (or any other habitat variable) were detected.

## Discussion

Organic farming benefitted spiders and carabid beetles, less consistently for the latter. The most marked effect was for pre-harvest hunting spiders in the cropped area. This guild includes the lycosids, which mainly disperse by walking. For almost all pairs of farms, we observed (before harvest) more hunting spiders to be captured on the organic farm of the pair (Fig 1b), and a similar pattern for species density. No effects of farming system were detectable for web-building spiders, many of which have high dispersal abilities (for example, dispersing by ballooning), suggesting that local impacts of pesticides, for example, on conventional farms are buffered by rapid recolonisation. A benefit of organic farming to spiders, restricted to hunting spiders, was also observed in Germany [42], and Schmidt et al. [41] reported similar system effects for two taxa of spiders which walked into fields from overwintering sites, and which were not observed for ballooning spiders.

The greater effect of organic farming on hunting spiders in more complex landscapes was unexpected. In the cropped area, it resulted from the presence of an upward trend in activity density and species density of hunting spiders in more arable landscapes on conventional farms but not organic farms. This is counter to the effect observed by Schmidt et al. [41] for abundance, who observed more spiders on conventional farms in complex landscapes, but no such effect on organic farms. It is also counter to the effect reported by Batary et al. [42] where hunting spider abundance was lower in more intensively managed landscapes, an effect which they found to be present on both organic and conventional farms, but to be more marked on conventional farms (though a formal test for system-landscape interaction does not appear to have been carried out, and the effect size is not reported). Schmidt et al. [41] suggested that non-crop habitats in the landscape increased local species richness of spiders, so that larger species pools are sustained in complex landscapes, where there is higher availability of refuge and overwintering habitats. Colonisation might therefore compensate for the inputs and disturbance on conventional fields. One possible explanation for the trend we observed (consistent over two years) could be an increasing reliance on the cropped area for resources in arable-dominated conventionally farmed landscapes, a trend not observed on organic crops where abundance and diversity were independent of landscape complexity. Agrobiont species—those which dominate arable landscapes [60]—appear adapted to disturbed habitats [61]. Female spiders of one agribiont genus, *Pardosa* (Lycosidae), which are hunting spiders, were found to be in better condition in landscapes dominated by arable crops [62]. Schmidt et al. [63] found that, while most spider species in arable fields benefited from non-crop habitats in the surrounding landscape, some arable species declined when landscapes became too dominated by non-crop habitats.

In the field boundary, the farming system effect also tended to be larger in simple landscapes, both before and after harvest (Fig 1c–1f). This outcome did not emerge from the same trends as were observed in the cropped area. In the boundary, activity density and species density declined with landscape homogeneity on organic farms, but were not affected by landscape on conventional farms. This is consistent with a landscape refuge effect such as suggested by Schmidt et al. [41], but only for organic farm boundaries. Lower quality boundaries on conventional farms, and fewer potential colonists in a homogenous landscape, may be one possible explanation for this effect.

We found no evidence that boundary or hedge characteristics (Table 1) were related to the observed farming system effects. For hunting spiders, the between-system difference was at least partly related to plant species richness within the crop, and there was a positive trend in hunting spider species density with plant species richness among conventional farms. The absence of herbicides in organic fields, resulting in larger non-crop plant populations, and more herbivorous prey, is an obvious plausible explanation [42]. The lack of insecticides is also likely to be at least partly responsible, if not a complete explanation (in Schmidt et al. [41], a positive effect of organic farming on spider density was found before any insecticides were applied). Agrochemical application is clearly confounded with management system. A link between the use of agrochemicals and reduced abundance of predators is well established. For example, in another study, the densities of linyphiid spiders where full pesticide inputs were applied were found to be around 47% of those in reduced-input areas [64]. Our study supports the *a priori* expectation that the farming system effect on biodiversity is linked to pesticide application.

The effect of organic farming on carabids was less consistent than for spiders. Previous work has shown that, at the farm-scale, the distribution of field and boundary overwintering carabid species shows considerable variation both within fields and boundaries [65]. Effects often vary between species and studies [40]. In their study in Sweden, Weibull et al. [37]) found that richness of carabids was higher on conventional farms; they suggested that the use of inorganic fertilisers and the higher level of plant production on conventional farms may have contributed to this result. In our study, there were more carabids in the cropped area of organic farms before harvest. Weedy areas are attractive to carabid beetles as sources of seed and invertebrate food, and organic farming may permit species more usually confined to field margins to persist in cropped areas [66]. Batary et al. [42] observed a stronger farming system effect for non-carnivore carabids. They also observed, as we did, that there was no positive effect of plant species richness within farming system.

Carabids may be less sensitive to structural diversity at the field scale. The quality and quantity of more permanent landscape elements such as hedgerow networks, used for dispersal and overwintering, may be more important [65]. We found a clear tendency for fewer carabid species to occur in simpler landscapes. This was contrary to Winquist et al. [40] who did not detect a landscape effect on carabid species richness but did observe more individuals in simpler (more arable) landscapes. The tendency we observed for lower activity density and species density of carabids in simple landscapes on both organic and conventional farms supports the idea that greater availability of overwintering sites and dispersal routes is important. Purtauf et al. [67], for example, found that species richness and activity density of carabids increased with percent cover of grassland in the surrounding landscape; in particular, they concluded that the activity density of spring breeders on organic fields benefited from the increased availability of overwintering habitats in their close surroundings. The lack of agreement between some studies on the effects of organic farming may also indicate a disparity in farming activities by farming system. Of the large-scale studies of the impacts of organic farming that are reported in the literature, most are from European countries other than the UK; our study is the largest scale UK study of which we are aware. Organic farming is not the same across Europe. For example, the size of organic holdings and the proportion of arable, livestock or horticulture that are organic differs among Member States and regions, depending on various factors such as the technical capabilities related to organic production, the support that different countries provide and the structure of consumer demand [68], all of which may affect the impacts on biodiversity of organic farming.

There was little evidence that the observed farming system differences for spiders or carabid beetles were explained by non-crop habitat differences (in hedgerow or boundary quality). So the answer to one of our main questions—can the farming system difference be explained by differences in non-crop habitats?—is ‘no’. While the quality of the uncropped habitat did vary between farming systems, with larger and more sympathetically managed hedgerows occurring on our sample of organic farms [20], we did not detect field level effects. Pywell et al. [69] concluded that hedgerows, rather than uncropped field boundary habitats, provided the highest quality overwintering habitat for invertebrates, including staphylinid and carabid beetles, and spiders and Brooks et al. [70] reported that carabid declines were less severe where hedgerows are managed for conservation. The absence of a field level link between hedgerows and carabids in our study is therefore surprising. Measures of activity provided by pitfall traps may be too crude for detecting an impact on carabid assemblages [71], and analyses of community structure will pursue this issue. A future area of focus for our data would be to classify carabids according to wing morphology, since dispersal ability is related to hindwing length (Gutierrez and Menendez 1997)[72].

## Conclusions and Applied Recommendations

Our results suggest that the farm system differences in the cropped area were probably attributable to crop management differences—including reduced inputs of pesticides (both herbicides and insecticides) and fertilisers and, for hunting spider activity density, plant species richness. Better targeting of pesticide use within conventional farms, and the possibility of field edge management with reduced pesticide inputs (‘conservation headlands’) could bring benefits; although little is known of how the scale over which such measures are put into place, and the length of time they are in place, affects the biodiversity benefits that accrue.

What are the main policy messages from our work bearing in mind that the EU is revising the nature of organic agriculture, so called 'uniformity of organic rules for farmers' across Europe to increase consumer confidence by harmonising permitted activities? The study showed that benefits of organic farming for biodiversity may be particularly marked for less mobile invertebrates. Which elements of organic farming can be transferred into conventional systems for biodiversity enhancement remains uncertain. We found landscape and farming system to interact in their effects, highlighting the importance of developing strategies for managing farmland at the landscape-scale for most effective conservation of biodiversity (e.g. in the UK, organic farms are concentrated in the more heterogeneous landscapes of south-western England where our results, for invertebrates at least, indicate the benefit of organic farming to biodiversity are likely to be greatest). The total area of organic farms relative to conventional is small (currently *ca*. 5.4% of European Union farmland is organic: [68]). If the benefit of organic farming is greater in some landscapes types, policy makers aiming to encourage conversion might need to consider regional targeting. Although the new EU agricultural policy has been criticised for not safeguarding farmland biodiversity sufficiently [12], it may also present an opportunity, in that it allows greater flexibility at a national level, including increased budgets for organic farming. At least this may allow the promotion of low intensity farming in landscapes where it may have the greatest benefit to biodiversity.

## Supporting Information

S1 Table Full model selection output for response variables, including model weights and model averaged parameter estimates. (DOCX)
