# Supplementary material for: Organic Farming: Biodiversity Impacts Can Depend on Dispersal Characteristics and Landscape Context
Source: PLoS One. 2015 Aug 26;10(8):e0135921. doi: 10.1371/journal.pone.0135921 (PMC4550245; doi:10.1371/journal.pone.0135921)
Supplement: S1 Table — (DOCX) [file pone.0135921.s001.docx]

Table S1. Full model selection output for response variables of interest, including model weights and model averaged parameter estimates.

SPIDERS – hunters. Before harvest abundance in winter wheat cropped area 2002-2003

Predictor variables:

1- arabkm1z (arable in 1 km x 1 km square around farm)

2- arabkm3z (arable in 3 km x 3 km square around farm)

3- calyear (calendar year , 2002,2003)

4-system (organic, conventional)

5-woodkm1z (woodland in 1 km x 1 km square around farm)

6-woodkm3z (woodland in 3 km x 3 km square around farm)

Interaction terms:

7-arabkm1z:system

8-arabkm3z:system

9-system:woodkm1z

10-system:woodkm3z

**Model summary:**

**Deviance AICc Delta Weight**

**2+3+4+8 206.13 221.18 0.00 0.33**

**2+3+4+5+8 204.99 222.36 1.18 0.18**

**2+3+4+6+8 205.67 223.04 1.86 0.13**

**2+3+4+6+8+10 204.58 224.31 3.13 0.07**

**2+3+4+5+8+9 204.64 224.37 3.18 0.07**

**1+3+4+7 209.34 224.40 3.22 0.07**

**1+3+4+5+7 208.17 225.55 4.36 0.04**

**1+3+4+6+7 209.33 226.70 5.52 0.02**

**1+3+4+6+7+10 207.76 227.49 6.31 0.01**

**1+3+4+5+7+9 208.05 227.78 6.60 0.01**

**3+4 217.58 228.13 6.95 0.01**

**2+3+4 215.74 228.52 7.34 0.01**

**2+3+4+6+10 211.72 229.10 7.91 0.01**

**3+4+6+10 214.21 229.27 8.08 0.01**

**2+3+4+5 214.63 229.69 8.50 0.00**

**3+4+5 216.94 229.73 8.54 0.00**

**1+3+4 217.06 229.84 8.66 0.00**

**2+3+4+6 215.02 230.08 8.90 0.00**

**3+4+6 217.45 230.23 9.05 0.00**

**2+3+4+5+9 213.22 230.59 9.41 0.00**

**3+4+5+9 215.58 230.64 9.45 0.00**

**1+3+4+6+10 213.52 230.90 9.71 0.00**

**1+3+4+5 216.04 231.10 9.91 0.00**

**1+3+4+6 216.79 231.84 10.66 0.00**

**1+3+4+5+9 214.91 232.28 11.10 0.00**

**3 244.42 252.78 31.60 0.00 Null model (calendar year only)**

**3+5 242.83 253.38 32.20 0.00**

**2+3 243.45 254.00 32.82 0.00**

**2+3+5 241.31 254.09 32.91 0.00**

**3+6 243.78 254.34 33.15 0.00**

**2+3+6 242.08 254.87 33.68 0.00**

**1+3 244.42 254.97 33.79 0.00**

**1+3+5 242.73 255.52 34.33 0.00**

**1+3+6 243.75 256.54 35.36 0.00**

**Model-averaged coefficients:**

**Coefficient SE z value Pr(>|z|)**

**(Intercept) 1.40476 0.10815 12.989 < 2e-16 *****

**arabkm1z 0.15685 0.08131 1.929 0.05373 .**

**arabkm3z 0.21991 0.08027 2.740 0.00615 ****

**calyear2003 -0.33825 0.14010 2.414 0.01576 ***

**systemO 0.57811 0.09260 6.243 < 2e-16 *****

**woodkm1z 0.05350 0.06745 0.793 0.42765**

**woodkm3z 0.01539 0.07388 0.208 0.83496**

**arabkm1z:systemO -0.29758 0.10548 2.821 0.00478 ****

**arabkm3z:systemO -0.30570 0.09636 3.172 0.00151 ****

**systemO:woodkm1z 0.06478 0.10822 0.599 0.54945**

**systemO:woodkm3z 0.13157 0.11123 1.183 0.23688**

Landscape effects and Organic Farms only:

**Model summary:**

**Deviance AICc Delta Weight**

**3 24.29 119.58 0.00 0.32**

**3+4 24.03 121.28 1.70 0.14**

**3+5 24.05 121.34 1.76 0.13**

**1+3 24.15 121.57 1.99 0.12**

**2+3 24.20 121.70 2.12 0.11**

**1+3+4 23.96 123.54 3.96 0.04**

**2+3+4 24.00 123.62 4.05 0.04**

**1+3+5 24.01 123.64 4.06 0.04**

**2+3+5 24.04 123.72 4.14 0.04**

**Variables:**

**1 2 3 4 5**

**arabkm1z arabkm3z calyear woodkm1z woodkm3z**

**Model-averaged coefficients:**

**Coefficient SE Adjusted SE z value Pr(>|z|)**

**(Intercept) 2.02052 0.12407 0.12690 15.922 <2e-16 *****

**arabkm1z -0.04264 0.09206 0.09417 0.453 0.6507**

**arabkm3z -0.03059 0.09258 0.09470 0.323 0.7466**

**calyear2003 -0.38336 0.17713 0.18118 2.116 0.0344 ***

**woodkm1z 0.06532 0.09081 0.09290 0.703 0.4820**

**woodkm3z 0.06143 0.09235 0.09447 0.650 0.5156**

Landscape effects and Conventional Farms only:

**Model summary:**

**Deviance AICc Delta Weight**

**2+3 20.22 111.46 0.00 0.42**

**1+3 20.87 113.27 1.81 0.17**

**2+3+5 20.14 113.63 2.17 0.14**

**2+3+4 20.18 113.75 2.29 0.13**

**1+3+4 20.70 115.19 3.73 0.06**

**1+3+5 20.87 115.67 4.21 0.05**

**3 23.70 118.18 6.72 0.01**

**3+5 23.65 120.40 8.94 0.00**

**3+4 23.68 120.46 9.00 0.00**

**Variables:**

**1 2 3 4 5**

**arabkm1z arabkm3z calyear woodkm1z woodkm3z**

**Model-averaged coefficients:**

**Coefficient SE Adjusted SE z value Pr(>|z|)**

**(Intercept) 1.41013 0.11532 0.11797 11.953 < 2e-16 *****

**arabkm1z 0.23075 0.08557 0.08753 2.636 0.00839 ****

**arabkm3z 0.25417 0.08366 0.08559 2.970 0.00298 ****

**calyear2003 -0.30582 0.16542 0.16923 1.807 0.07074 .**

**woodkm1z 0.03671 0.08697 0.08893 0.413 0.67974**

**woodkm3z 0.02676 0.08862 0.09056 0.296 0.76761**

**---**

SPIDERS – hunters. Before harvest species richness in winter wheat cropped area 2002-2003

**Model summary:**

**Deviance AICc Delta Weight**

**2+3+4+6+8 62.29 79.66 0.00 0.22**

**2+3+4+8 65.43 80.48 0.83 0.14**

**2+3+4+5+8 63.18 80.56 0.90 0.14**

**1+3+4+5+7 63.74 81.11 1.45 0.10**

**1+3+4+6+7 63.95 81.32 1.67 0.09**

**1+3+4+7 66.27 81.33 1.67 0.09**

**2+3+4+6+8+10 62.28 82.01 2.35 0.07**

**2+3+4+5+8+9 63.18 82.91 3.25 0.04**

**1+3+4+5+7+9 63.72 83.45 3.79 0.03**

**1+3+4+6+7+10 63.93 83.66 4.00 0.03**

**3+4+6 72.97 85.75 6.09 0.01**

**3+4+5 74.46 87.25 7.59 0.00**

**3+4+6+10 72.21 87.27 7.61 0.00**

**3+4 76.91 87.47 7.81 0.00**

**2+3+4+6 72.96 88.01 8.36 0.00**

**1+3+4+6 72.96 88.02 8.36 0.00**

**3+4+5+9 73.83 88.89 9.23 0.00**

**2+3+4 76.60 89.39 9.73 0.00**

**2+3+4+5 74.39 89.45 9.79 0.00**

**1+3+4 76.68 89.47 9.81 0.00**

**1+3+4+5 74.46 89.51 9.86 0.00**

**2+3+4+6+10 72.20 89.57 9.91 0.00**

**1+3+4+6+10 72.21 89.59 9.93 0.00**

**2+3+4+5+9 73.77 91.15 11.49 0.00**

**1+3+4+5+9 73.82 91.19 11.53 0.00**

**3+6 99.35 109.90 30.24 0.00**

**3+5 100.56 111.11 31.45 0.00**

**1+3+6 98.67 111.45 31.79 0.00**

**2+3+6 99.31 112.10 32.44 0.00**

**1+3+5 99.94 112.73 33.07 0.00**

**2+3+5 100.30 113.08 33.43 0.00**

**3 104.75 113.12 33.46 0.00 Null model**

**1+3 103.01 113.57 33.91 0.00**

**2+3 103.93 114.48 34.82 0.00**

**Model-averaged coefficients:**

**Coefficient SE z value Pr(>|z|)**

**(Intercept) 1.2855039 0.0575520 22.336 < 2e-16 *****

**arabkm1z 0.0636565 0.0424647 1.499 0.13386**

**arabkm3z 0.0672526 0.0432666 1.554 0.12009**

**calyear2003 -0.2417787 0.0744328 3.248 0.00116 ****

**systemO 0.3096354 0.0505193 6.129 < 2e-16 *****

**woodkm1z 0.0488443 0.0351407 1.390 0.16454**

**woodkm3z 0.0557937 0.0350121 1.594 0.11104**

**arabkm1z:systemO -0.1849083 0.0565018 3.273 0.00107 ****

**arabkm3z:systemO -0.1801228 0.0518243 3.476 0.00051 *****

**systemO:woodkm1z 0.0006463 0.0587555 0.011 0.99122**

**systemO:woodkm3z 0.0030720 0.0602899 0.051 0.95936**

Landscape effects and Organic Farms only:

**Model summary:**

**Deviance AICc Delta Weight**

**2+3 6.75 48.94 0.00 0.22**

**1+3 6.81 49.43 0.48 0.18**

**2+3+4 6.64 50.38 1.44 0.11**

**2+3+5 6.67 50.61 1.67 0.10**

**1+3+4 6.69 50.83 1.89 0.09**

**1+3+5 6.71 50.95 2.01 0.08**

**3+5 7.00 50.96 2.01 0.08**

**3 7.30 51.09 2.15 0.08**

**3+4 7.04 51.33 2.39 0.07**

**Variables:**

**1 2 3 4 5**

**arabkm1z arabkm3z calyear woodkm1z woodkm3z**

**Model-averaged coefficients:**

**Coefficient SE Adjusted SE z value Pr(>|z|)**

**(Intercept) 1.62846 0.06633 0.06785 24.001 < 2e-16 *****

**arabkm1z -0.08682 0.04930 0.05041 1.722 0.08503 .**

**arabkm3z -0.09271 0.04919 0.05031 1.843 0.06534 .**

**calyear2003 -0.26584 0.09490 0.09707 2.739 0.00617 ****

**woodkm1z 0.05259 0.04972 0.05084 1.034 0.30096**

**woodkm3z 0.05362 0.05211 0.05324 1.007 0.31383**

Landscape effects and conventional farms only:

**Model summary:**

**Deviance AICc Delta Weight**

**2+3+5 5.79 42.61 0.00 0.19**

**1+3+5 5.83 43.01 0.41 0.15**

**1+3 6.09 43.08 0.47 0.15**

**3 6.40 43.54 0.93 0.12**

**2+3 6.15 43.65 1.04 0.11**

**3+5 6.19 44.00 1.39 0.09**

**1+3+4 5.94 44.08 1.47 0.09**

**2+3+4 6.07 45.30 2.70 0.05**

**3+4 6.36 45.53 2.92 0.04**

**Variables:**

**1 2 3 4 5**

**arabkm1z arabkm3z calyear woodkm1z woodkm3z**

**Model-averaged coefficients:**

**Coefficient SE Adjusted SE z value Pr(>|z|)**

**(Intercept) 1.27771 0.06286 0.06430 19.872 <2e-16 *****

**arabkm1z 0.08095 0.04617 0.04722 1.714 0.0865 .**

**arabkm3z 0.07909 0.04703 0.04807 1.645 0.0999 .**

**calyear2003 -0.21656 0.09044 0.09250 2.341 0.0192 ***

**woodkm1z 0.04324 0.04806 0.04911 0.880 0.3786**

**woodkm3z 0.07367 0.04658 0.04762 1.547 0.1219**

SPIDERS – Web builders. Before harvest abundance in winter wheat cropped area 2002-2003

**Model summary:**

**Deviance AICc Delta Weight**

**1+3 232.64 243.20 0.00 0.14**

**3 236.00 244.37 1.17 0.08 Null model (calendar year only)**

**2+3 233.85 244.41 1.21 0.08**

**3+5 233.92 244.47 1.28 0.07**

**1+3+5 231.75 244.53 1.33 0.07**

**1+3+4 232.37 245.16 1.96 0.05**

**2+3+5 232.39 245.18 1.98 0.05**

**1+3+6 232.40 245.19 1.99 0.05**

**3+4 234.84 245.40 2.20 0.05**

**3+6 235.27 245.83 2.63 0.04**

**2+3+4 233.16 245.95 2.75 0.03**

**3+4+5 233.28 246.07 2.87 0.03**

**2+3+6 233.69 246.47 3.27 0.03**

**1+3+4+5 231.58 246.64 3.44 0.02**

**1+3+4+7 231.82 246.88 3.68 0.02**

**3+4+5+9 231.93 246.99 3.79 0.02**

**2+3+4+5 232.00 247.06 3.86 0.02**

**3+4+6 234.36 247.14 3.95 0.02**

**1+3+4+6 232.18 247.23 4.03 0.02**

**1+3+4+5+9 230.38 247.75 4.56 0.01**

**2+3+4+5+9 230.48 247.85 4.66 0.01**

**2+3+4+6 233.06 248.12 4.92 0.01**

**2+3+4+8 233.16 248.21 5.02 0.01**

**1+3+4+5+7 231.05 248.42 5.23 0.01**

**1+3+4+6+7 231.52 248.90 5.70 0.01**

**3+4+6+10 234.10 249.15 5.95 0.01**

**2+3+4+5+8 232.00 249.37 6.17 0.01**

**1+3+4+6+10 232.04 249.41 6.22 0.01**

**1+3+4+5+7+9 230.21 249.94 6.75 0.00**

**2+3+4+5+8+9 230.34 250.07 6.87 0.00**

**2+3+4+6+10 232.86 250.23 7.03 0.00**

**2+3+4+6+8 233.05 250.43 7.23 0.00**

**1+3+4+6+7+10 231.14 250.87 7.67 0.00**

**2+3+4+6+8+10 232.85 252.58 9.38 0.00**

**Model-averaged coefficients:**

**Coefficient SE z value Pr(>|z|)**

**(Intercept) 1.90375 0.12580 15.133 <2e-16 *****

**arabkm1z 0.11238 0.06996 1.606 0.108**

**arabkm3z 0.09874 0.07714 1.280 0.201**

**calyear2003 0.24845 0.17319 1.435 0.151**

**systemO -0.06572 0.09640 0.682 0.495**

**woodkm1z -0.08488 0.07282 1.166 0.244**

**woodkm3z -0.03392 0.06826 0.497 0.619**

**arabkm1z:systemO -0.07790 0.10728 0.726 0.468**

**arabkm3z:systemO 0.01215 0.10046 0.121 0.904**

**systemO:woodkm1z 0.12669 0.11006 1.151 0.250**

**systemO:woodkm3z -0.05113 0.10895 0.469 0.639**

SPIDERS – Web builders. Before harvest species richness in winter wheat cropped area 2002-2003

**Model summary:**

**Deviance AICc Delta Weight**

**3+5 85.87 96.43 0.00 0.12**

**1+3 86.49 97.04 0.61 0.09**

**1+3+5 84.58 97.36 0.93 0.07**

**3 89.12 97.48 1.06 0.07 Null model**

**2+3+5 85.04 97.82 1.40 0.06**

**3+4+5 85.24 98.02 1.59 0.05**

**2+3 87.55 98.11 1.68 0.05**

**3+6 87.72 98.27 1.84 0.05**

**3+4 87.88 98.44 2.01 0.04**

**1+3+6 85.76 98.54 2.11 0.04**

**1+3+4 86.03 98.82 2.39 0.04**

**3+4+5+9 83.94 99.00 2.57 0.03**

**1+3+4+5 84.29 99.35 2.92 0.03**

**2+3+4 86.68 99.46 3.03 0.03**

**3+4+6 86.79 99.57 3.14 0.02**

**2+3+4+5 84.55 99.61 3.18 0.02**

**2+3+6 86.88 99.66 3.24 0.02**

**1+3+4+6 85.38 100.44 4.01 0.02**

**1+3+4+5+9 83.10 100.48 4.05 0.02**

**2+3+4+5+9 83.16 100.53 4.10 0.02**

**3+4+6+10 85.52 100.57 4.15 0.02**

**1+3+4+7 86.00 101.05 4.62 0.01**

**2+3+4+6 86.13 101.19 4.76 0.01**

**2+3+4+8 86.28 101.34 4.91 0.01**

**2+3+4+5+8 84.20 101.57 5.14 0.01**

**1+3+4+5+7 84.25 101.63 5.20 0.01**

**1+3+4+6+10 84.38 101.75 5.32 0.01**

**2+3+4+5+8+9 82.34 102.07 5.64 0.01**

**2+3+4+6+10 84.98 102.35 5.92 0.01**

**1+3+4+5+7+9 82.80 102.53 6.10 0.01**

**1+3+4+6+7 85.38 102.75 6.32 0.01**

**2+3+4+6+8 85.78 103.15 6.72 0.00**

**1+3+4+6+7+10 84.33 104.06 7.64 0.00**

**2+3+4+6+8+10 84.91 104.65 8.22 0.00**

**Model-averaged coefficients:**

**Coefficient SE z value Pr(>|z|)**

**(Intercept) 1.55635 0.06048 25.732 <2e-16 *****

**arabkm1z 0.04600 0.03774 1.219 0.223**

**arabkm3z 0.03578 0.04023 0.889 0.374**

**calyear2003 0.16837 0.08114 2.075 0.038 ***

**systemO -0.04304 0.05580 0.771 0.441**

**woodkm1z -0.06032 0.03941 1.531 0.126**

**woodkm3z -0.02821 0.03845 0.734 0.463**

**arabkm1z:systemO 0.01252 0.06275 0.200 0.842**

**arabkm3z:systemO 0.03768 0.05837 0.646 0.519**

**systemO:woodkm1z 0.07226 0.06190 1.167 0.243**

**systemO:woodkm3z -0.06531 0.06104 1.070 0.285**

SPIDERS – hunters. Before harvest abundance in winter wheat field boundary 2002-2003

**Model summary:**

**Deviance AICc Delta Weight**

**2+3+4+5+8 247.74 265.12 0.00 0.11**

**1+3+4+5+7 247.75 265.13 0.01 0.11**

**2+3+4+8 250.23 265.28 0.17 0.10**

**2+3+4+6+8 248.08 265.45 0.34 0.10**

**1+3+4+6+7 248.40 265.77 0.65 0.08**

**1+3+4+7 251.30 266.36 1.24 0.06**

**2+3+5 253.61 266.40 1.28 0.06**

**2+3+6 253.93 266.71 1.59 0.05**

**2+3 256.64 267.19 2.08 0.04**

**2+3+4+5+8+9 247.65 267.38 2.26 0.04**

**1+3+4+5+7+9 247.75 267.48 2.37 0.03**

**2+3+4+6+8+10 248.07 267.80 2.69 0.03**

**1+3+4+6+7+10 248.38 268.11 3.00 0.03**

**2+3+4+5 253.21 268.27 3.15 0.02**

**2+3+4+6 253.37 268.43 3.31 0.02**

**2+3+4 255.82 268.60 3.49 0.02**

**3+6 258.48 269.03 3.92 0.02**

**3+5 259.29 269.85 4.73 0.01**

**2+3+4+5+9 252.51 269.89 4.77 0.01**

**2+3+4+6+10 252.65 270.03 4.91 0.01**

**3+4+6 257.52 270.31 5.19 0.01**

**1+3+6 257.69 270.47 5.36 0.01**

**3+4+5 258.46 271.25 6.13 0.01**

**1+3+5 258.76 271.55 6.43 0.00**

**3+4+6+10 256.56 271.62 6.50 0.00**

**1+3+4+6 257.11 272.16 7.05 0.00**

**3 264.04 272.41 7.29 0.00 Null model**

**3+4+5+9 257.56 272.62 7.50 0.00**

**1+3 262.30 272.86 7.74 0.00**

**3+4 262.34 272.89 7.77 0.00**

**1+3+4+5 258.21 273.26 8.15 0.00**

**1+3+4+6+10 256.29 273.66 8.54 0.00**

**1+3+4 261.40 274.19 9.07 0.00**

**1+3+4+5+9 257.26 274.63 9.52 0.00**

**Model-averaged coefficients:**

**Coefficient SE z value Pr(>|z|)**

**(Intercept) 1.65541 0.12920 12.813 < 2e-16 *****

**arabkm1z 0.10024 0.10223 0.980 0.32684**

**arabkm3z -0.11566 0.10832 1.068 0.28567**

**calyear2003 -0.41926 0.16877 2.484 0.01298 ***

**systemO 0.08471 0.11468 0.739 0.46012**

**woodkm1z 0.12167 0.08003 1.520 0.12841**

**woodkm3z 0.12229 0.07973 1.534 0.12505**

**arabkm1z:systemO -0.39682 0.12620 3.144 0.00166 ****

**arabkm3z:systemO -0.27620 0.11843 2.332 0.01969 ***

**systemO:woodkm1z 0.03758 0.13519 0.278 0.78100**

**systemO:woodkm3z 0.03636 0.13779 0.264 0.79189**

SPIDERS – hunters. Before harvest abundance in winter wheat field boundary 2002-2003

**Organic only**

**Model summary:**

**Deviance AICc Delta Weight**

**2+3 32.71 138.87 0.00 0.34**

**2+3+4 31.71 139.50 0.63 0.25**

**2+3+5 31.96 139.95 1.08 0.20**

**1+3 34.37 141.69 2.82 0.08**

**1+3+4 33.24 142.20 3.33 0.06**

**1+3+5 33.30 142.29 3.42 0.06**

**3+5 38.11 147.58 8.71 0.00**

**3+4 38.97 148.85 9.98 0.00**

**3 41.94 150.73 11.86 0.00**

**Variables:**

**1 2 3 4 5**

**arabkm1z arabkm3z calyear woodkm1z woodkm3z**

**Model-averaged coefficients:**

**Coefficient SE Adjusted SE z value Pr(>|z|)**

**(Intercept) 1.7694 0.1459 0.1492 11.856 < 2e-16 *****

**arabkm1z -0.3407 0.1120 0.1145 2.976 0.002920 ****

**arabkm3z -0.3840 0.1092 0.1116 3.441 0.000579 *****

**calyear2003 -0.3965 0.2095 0.2143 1.850 0.064281 .**

**woodkm1z 0.1408 0.1078 0.1103 1.276 0.201826**

**woodkm3z 0.1329 0.1142 0.1168 1.138 0.255126**

SPIDERS – hunters. Before harvest abundance in winter wheat field boundary 2002-2003

**Conventional only**

**Model summary:**

**Deviance AICc Delta Weight**

**3+5 30.93 135.67 0.00 0.23**

**3 32.72 136.58 0.90 0.15**

**3+4 31.54 136.79 1.12 0.13**

**1+3+5 30.29 136.90 1.23 0.13**

**1+3+4 30.41 137.13 1.46 0.11**

**2+3+5 30.87 137.97 2.30 0.07**

**1+3 32.28 138.13 2.45 0.07**

**2+3 32.38 138.30 2.62 0.06**

**2+3+4 31.38 138.92 3.24 0.05**

**Variables:**

**1 2 3 4 5**

**arabkm1z arabkm3z calyear woodkm1z woodkm3z**

**Model-averaged coefficients:**

**Coefficient SE Adjusted SE z value Pr(>|z|)**

**(Intercept) 1.64209 0.14283 0.14609 11.240 <2e-16 *****

**arabkm1z 0.11934 0.10769 0.11007 1.084 0.2782**

**arabkm3z -0.05423 0.10718 0.10959 0.495 0.6207**

**calyear2003 -0.43329 0.20542 0.21007 2.063 0.0392 ***

**woodkm1z 0.16222 0.10717 0.10953 1.481 0.1386**

**woodkm3z 0.18220 0.10312 0.10549 1.727 0.0841 .**

SPIDERS – hunters. Before harvest species richness in winter wheat field boundary 2002-2003

**Model summary:**

**Deviance AICc Delta Weight**

**2+3 130.43 140.99 0.00 0.12**

**2+3+5 128.41 141.19 0.21 0.11**

**2+3+6 128.48 141.26 0.28 0.11**

**2+3+4+8 126.54 141.60 0.61 0.09**

**2+3+4+5+8 124.88 142.25 1.26 0.07**

**2+3+4+6+8 124.97 142.34 1.35 0.06**

**2+3+4 129.94 142.73 1.74 0.05**

**2+3+4+6 128.16 143.22 2.24 0.04**

**2+3+4+5 128.18 143.24 2.25 0.04**

**3+6 133.19 143.74 2.76 0.03**

**1+3+4+5+7 126.61 143.98 3.00 0.03**

**1+3+4+6+7 126.72 144.09 3.11 0.03**

**1+3+4+7 129.29 144.35 3.36 0.02**

**2+3+4+6+8+10 124.66 144.39 3.40 0.02**

**2+3+4+5+8+9 124.87 144.60 3.62 0.02**

**3+5 134.15 144.70 3.72 0.02**

**2+3+4+5+9 127.96 145.33 4.34 0.01**

**3+4+6 132.55 145.33 4.35 0.01**

**2+3+4+6+10 128.16 145.53 4.55 0.01**

**1+3+6 132.75 145.53 4.55 0.01**

**3 137.69 146.06 5.07 0.01 Null model**

**1+3+4+6+7+10 126.45 146.18 5.19 0.01**

**1+3+4+5+7+9 126.58 146.31 5.32 0.01**

**3+4+5 133.58 146.36 5.38 0.01**

**1+3+5 133.86 146.65 5.66 0.01**

**3+4 136.49 147.04 6.06 0.01**

**1+3 136.57 147.12 6.14 0.01**

**1+3+4+6 132.34 147.39 6.41 0.01**

**3+4+6+10 132.50 147.56 6.57 0.00**

**3+4+5+9 133.23 148.29 7.30 0.00**

**1+3+4+5 133.46 148.51 7.53 0.00**

**1+3+4 135.91 148.69 7.71 0.00**

**1+3+4+6+10 132.31 149.68 8.69 0.00**

**1+3+4+5+9 133.09 150.46 9.48 0.00**

**Model-averaged coefficients:**

**Coefficient SE z value Pr(>|z|)**

**(Intercept) 1.37957 0.07388 18.674 <2e-16 *****

**arabkm1z 0.02969 0.06381 0.465 0.6418**

**arabkm3z -0.09421 0.05688 1.656 0.0977 .**

**calyear2003 -0.17142 0.09858 1.739 0.0820 .**

**systemO 0.03947 0.06647 0.594 0.5527**

**woodkm1z 0.05907 0.04497 1.314 0.1890**

**woodkm3z 0.06736 0.04623 1.457 0.1451**

**arabkm1z:systemO -0.18772 0.07396 2.538 0.0111 ***

**arabkm3z:systemO -0.12583 0.06874 1.831 0.0672 .**

**systemO:woodkm1z 0.01359 0.07790 0.174 0.8615**

**systemO:woodkm3z -0.02345 0.08013 0.293 0.7698**

SPIDERS – hunters. Before harvest species richness in winter wheat field boundary 2002-2003

**Organic Only**

**Model summary:**

**Deviance AICc Delta Weight**

**2+3 10.25 72.73 0.00 0.46**

**2+3+4 10.09 74.23 1.50 0.22**

**2+3+5 10.19 74.79 2.05 0.16**

**1+3 10.90 76.22 3.49 0.08**

**1+3+4 10.69 77.55 4.81 0.04**

**1+3+5 10.76 77.91 5.18 0.03**

**3+5 12.06 81.98 9.25 0.00**

**3+4 12.14 82.38 9.64 0.00**

**3 12.75 82.87 10.14 0.00**

**Variables:**

**1 2 3 4 5**

**arabkm1z arabkm3z calyear woodkm1z woodkm3z**

**Model-averaged coefficients:**

**Coefficient SE Adjusted SE z value Pr(>|z|)**

**(Intercept) 1.43216 0.08189 0.08377 17.095 < 2e-16 *****

**arabkm1z -0.17378 0.06224 0.06365 2.730 0.006331 ****

**arabkm3z -0.20559 0.06029 0.06166 3.334 0.000856 *****

**calyear2003 -0.17521 0.11743 0.12010 1.459 0.144604**

**woodkm1z 0.05747 0.06094 0.06234 0.922 0.356596**

**woodkm3z 0.04086 0.06476 0.06622 0.617 0.537167**

SPIDERS – hunters. Before harvest species richness in winter wheat field boundary 2002-2003

**Conventional Only**

**Model summary:**

**Deviance AICc Delta Weight**

**3+5 11.07 77.10 0.00 0.32**

**1+3+5 10.88 78.53 1.44 0.16**

**3 11.94 79.10 2.00 0.12**

**2+3+5 11.01 79.24 2.14 0.11**

**3+4 11.56 79.57 2.47 0.09**

**2+3 11.72 80.39 3.29 0.06**

**1+3+4 11.24 80.39 3.29 0.06**

**1+3 11.82 80.86 3.77 0.05**

**2+3+4 11.43 81.35 4.25 0.04**

**Variables:**

**1 2 3 4 5**

**arabkm1z arabkm3z calyear woodkm1z woodkm3z**

**Model-averaged coefficients:**

**Coefficient SE Adjusted SE z value Pr(>|z|)**

**(Intercept) 1.35574 0.08580 0.08776 15.448 <2e-16 *****

**arabkm1z 0.06123 0.06369 0.06513 0.940 0.3471**

**arabkm3z -0.04381 0.06476 0.06620 0.662 0.5081**

**calyear2003 -0.16527 0.12351 0.12631 1.309 0.1907**

**woodkm1z 0.08877 0.06449 0.06592 1.347 0.1781**

**woodkm3z 0.12580 0.06179 0.06321 1.990 0.0466 ***

SPIDERS – web builders. Before harvest abundance in winter wheat field boundary 2002-2003

**Model summary:**

**Deviance AICc Delta Weight**

**2+3 217.88 228.44 0.00 0.16**

**1+3 218.24 228.79 0.36 0.13**

**2+3+6 217.48 230.26 1.82 0.06**

**3 222.12 230.49 2.05 0.06 Null model**

**2+3+4 217.80 230.59 2.15 0.05**

**2+3+5 217.81 230.59 2.16 0.05**

**1+3+5 217.96 230.75 2.31 0.05**

**1+3+6 218.12 230.90 2.47 0.05**

**2+3+4+8 215.87 230.93 2.49 0.05**

**1+3+4 218.24 231.02 2.59 0.04**

**1+3+4+7 216.41 231.46 3.03 0.03**

**3+4 221.69 232.24 3.80 0.02**

**2+3+4+6 217.35 232.40 3.96 0.02**

**3+6 222.12 232.68 4.24 0.02**

**3+5 222.12 232.68 4.24 0.02**

**2+3+4+5 217.69 232.75 4.31 0.02**

**2+3+4+6+8 215.43 232.80 4.36 0.02**

**1+3+4+5 217.96 233.01 4.58 0.02**

**2+3+4+5+8 215.74 233.11 4.67 0.02**

**1+3+4+6 218.12 233.17 4.74 0.01**

**1+3+4+5+7 216.04 233.41 4.97 0.01**

**1+3+4+6+7 216.36 233.73 5.29 0.01**

**2+3+4+6+8+10 214.36 234.09 5.65 0.01**

**3+4+6 221.66 234.44 6.00 0.01**

**3+4+5 221.67 234.45 6.01 0.01**

**2+3+4+6+10 217.09 234.46 6.02 0.01**

**2+3+4+5+9 217.69 235.06 6.62 0.01**

**1+3+4+5+7+9 215.42 235.15 6.71 0.01**

**1+3+4+5+9 217.87 235.24 6.80 0.01**

**2+3+4+5+8+9 215.54 235.27 6.83 0.01**

**1+3+4+6+10 217.93 235.30 6.86 0.01**

**1+3+4+6+7+10 215.63 235.37 6.93 0.00**

**3+4+6+10 221.30 236.36 7.92 0.00**

**3+4+5+9 221.63 236.68 8.24 0.00**

**Model-averaged coefficients:**

**Coefficient SE z value Pr(>|z|)**

**(Intercept) 1.48562 0.11894 12.490 <2e-16 *****

**arabkm1z 0.13595 0.06813 1.995 0.0460 ***

**arabkm3z 0.16144 0.07539 2.141 0.0322 ***

**calyear2003 0.26844 0.16530 1.624 0.1044**

**systemO -0.02339 0.08841 0.265 0.7913**

**woodkm1z 0.02513 0.06386 0.394 0.6939**

**woodkm3z 0.03422 0.06582 0.520 0.6031**

**arabkm1z:systemO -0.13765 0.09795 1.405 0.1599**

**arabkm3z:systemO -0.13016 0.09042 1.439 0.1500**

**systemO:woodkm1z -0.03873 0.10519 0.368 0.7127**

**systemO:woodkm3z -0.07335 0.10409 0.705 0.4810**

SPIDERS – web builders. Before harvest species richness in winter wheat field boundary 2002-2003

**Model summary:**

**Deviance AICc Delta Weight**

**1+3 106.00 116.56 0.00 0.14**

**3 108.25 116.62 0.06 0.14 Null model**

**2+3 106.41 116.97 0.41 0.11**

**1+3+6 105.75 118.53 1.98 0.05**

**1+3+5 105.84 118.63 2.07 0.05**

**3+4 108.15 118.71 2.15 0.05**

**1+3+4 105.98 118.76 2.20 0.05**

**3+6 108.21 118.76 2.21 0.05**

**2+3+6 105.99 118.77 2.22 0.05**

**3+5 108.25 118.81 2.25 0.05**

**2+3+5 106.38 119.16 2.61 0.04**

**2+3+4 106.40 119.19 2.63 0.04**

**1+3+4+7 105.14 120.19 3.64 0.02**

**1+3+4+6 105.74 120.79 4.24 0.02**

**3+4+6 108.08 120.87 4.31 0.02**

**1+3+4+5 105.83 120.89 4.33 0.02**

**3+4+5 108.15 120.94 4.38 0.02**

**2+3+4+8 105.96 121.02 4.46 0.02**

**2+3+4+6 105.96 121.02 4.46 0.02**

**2+3+4+5 106.36 121.42 4.86 0.01**

**1+3+4+5+7 104.98 122.35 5.79 0.01**

**1+3+4+6+7 104.99 122.36 5.80 0.01**

**3+4+6+10 107.80 122.86 6.30 0.01**

**2+3+4+6+8 105.55 122.92 6.36 0.01**

**3+4+5+9 107.89 122.95 6.39 0.01**

**1+3+4+6+10 105.59 122.97 6.41 0.01**

**1+3+4+5+9 105.65 123.02 6.46 0.01**

**2+3+4+6+10 105.76 123.13 6.57 0.01**

**2+3+4+5+8 105.92 123.29 6.74 0.00**

**2+3+4+5+9 106.02 123.39 6.83 0.00**

**1+3+4+6+7+10 104.57 124.30 7.74 0.00**

**1+3+4+5+7+9 104.95 124.68 8.13 0.00**

**2+3+4+6+8+10 105.05 124.78 8.23 0.00**

**2+3+4+5+8+9 105.73 125.46 8.91 0.00**

**Model-averaged coefficients:**

**Coefficient SE z value Pr(>|z|)**

**(Intercept) 1.365516 0.069260 19.716 <2e-16 *****

**arabkm1z 0.062247 0.040508 1.537 0.1244**

**arabkm3z 0.062146 0.044022 1.412 0.1580**

**calyear2003 0.163926 0.096248 1.703 0.0885 .**

**systemO -0.004334 0.057065 0.076 0.9395**

**woodkm1z 0.005929 0.039360 0.151 0.8803**

**woodkm3z 0.019541 0.039563 0.494 0.6214**

**arabkm1z:systemO -0.057767 0.062932 0.918 0.3587**

**arabkm3z:systemO -0.039310 0.058735 0.669 0.5033**

**systemO:woodkm1z 0.029409 0.065285 0.450 0.6524**

**systemO:woodkm3z -0.032332 0.064285 0.503 0.6150**

SPIDERS – Hunters. After harvest abundance in winter wheat cropped area 2002-2003

**Model summary:**

**Deviance AICc Delta Weight**

**3 140.02 148.59 0.00 0.15 Null model**

**2+3 138.21 149.07 0.48 0.11**

**2+3+6 136.79 150.01 1.42 0.07**

**1+3 139.21 150.07 1.48 0.07**

**3+4 139.64 150.50 1.91 0.06**

**3+5 139.75 150.61 2.02 0.05**

**2+3+5 137.41 150.62 2.04 0.05**

**2+3+4 137.42 150.64 2.05 0.05**

**3+6 139.86 150.72 2.13 0.05**

**1+3+4 138.21 151.43 2.84 0.04**

**2+3+4+6 135.98 151.63 3.04 0.03**

**1+3+5 138.49 151.70 3.12 0.03**

**1+3+6 138.53 151.75 3.16 0.03**

**2+3+4+5 136.75 152.40 3.81 0.02**

**3+4+5 139.45 152.66 4.08 0.02**

**3+4+6 139.52 152.74 4.15 0.02**

**2+3+4+8 137.24 152.88 4.30 0.02**

**1+3+4+6 137.41 153.05 4.47 0.02**

**1+3+4+5 137.52 153.17 4.58 0.01**

**2+3+4+6+10 135.11 153.26 4.67 0.01**

**1+3+4+7 138.20 153.85 5.26 0.01**

**2+3+4+6+8 135.74 153.89 5.30 0.01**

**2+3+4+5+9 135.78 153.93 5.34 0.01**

**3+4+5+9 138.54 154.19 5.60 0.01**

**1+3+4+5+9 136.18 154.32 5.74 0.01**

**3+4+6+10 138.82 154.47 5.88 0.01**

**1+3+4+6+10 136.53 154.68 6.09 0.01**

**2+3+4+5+8 136.55 154.70 6.12 0.01**

**1+3+4+6+7 137.40 155.55 6.96 0.00**

**1+3+4+5+7 137.48 155.63 7.04 0.00**

**2+3+4+6+8+10 135.10 155.83 7.24 0.00**

**2+3+4+5+8+9 135.73 156.46 7.87 0.00**

**1+3+4+5+7+9 135.83 156.56 7.97 0.00**

**1+3+4+6+7+10 136.27 156.99 8.41 0.00**

**Model-averaged coefficients:**

**Coefficient SE z value Pr(>|z|)**

**(Intercept) 0.92301 0.11079 8.331 <2e-16 *****

**arabkm1z 0.08872 0.08080 1.098 0.272**

**arabkm3z 0.11466 0.07929 1.446 0.148**

**CALYEAR2003 -0.06536 0.13919 0.470 0.639**

**systemO 0.11818 0.14324 0.825 0.409**

**woodkm1z 0.06384 0.08268 0.772 0.440**

**woodkm3z 0.07858 0.08997 0.873 0.382**

**arabkm1z:systemO -0.03265 0.15276 0.214 0.831**

**arabkm3z:systemO 0.05611 0.14296 0.392 0.695**

**systemO:woodkm1z -0.15162 0.14086 1.076 0.282**

**systemO:woodkm3z -0.13132 0.14156 0.928 0.354**

SPIDERS – Hunters. After harvest species richness in winter wheat cropped area 2002-2003

**Model summary:**

**Deviance AICc Delta Weight**

**3 41.51 50.08 0.00 0.13 Null model**

**3+4 39.30 50.16 0.08 0.13**

**2+3+4 38.25 51.47 1.39 0.07**

**2+3 41.00 51.86 1.78 0.05**

**3+6 41.05 51.91 1.83 0.05**

**1+3+4 38.71 51.93 1.85 0.05**

**3+4+6 38.96 52.18 2.10 0.05**

**3+5 41.35 52.21 2.13 0.05**

**1+3 41.48 52.34 2.26 0.04**

**3+4+5 39.26 52.47 2.39 0.04**

**2+3+4+6 36.83 52.48 2.40 0.04**

**2+3+6 39.68 52.90 2.82 0.03**

**1+3+4+6 37.82 53.46 3.39 0.02**

**2+3+4+5 38.04 53.69 3.61 0.02**

**2+3+4+8 38.20 53.85 3.77 0.02**

**2+3+5 40.65 53.86 3.79 0.02**

**1+3+6 40.83 54.04 3.97 0.02**

**1+3+4+7 38.44 54.09 4.01 0.02**

**1+3+4+5 38.51 54.16 4.08 0.02**

**3+4+6+10 38.54 54.19 4.11 0.02**

**2+3+4+6+10 36.30 54.45 4.37 0.01**

**1+3+5 41.25 54.47 4.39 0.01**

**3+4+5+9 38.90 54.55 4.47 0.01**

**2+3+4+6+8 36.75 54.90 4.82 0.01**

**1+3+4+6+10 37.29 55.44 5.36 0.01**

**1+3+4+6+7 37.57 55.72 5.64 0.01**

**2+3+4+5+9 37.67 55.82 5.74 0.01**

**1+3+4+5+9 37.99 56.14 6.06 0.01**

**2+3+4+5+8 37.99 56.14 6.06 0.01**

**1+3+4+5+7 38.19 56.34 6.26 0.01**

**2+3+4+6+8+10 36.29 57.02 6.94 0.00**

**1+3+4+6+7+10 36.52 57.24 7.16 0.00**

**1+3+4+5+7+9 37.27 58.00 7.92 0.00**

**2+3+4+5+8+9 37.67 58.39 8.32 0.00**

**Model-averaged coefficients:**

**Coefficient SE z value Pr(>|z|)**

**(Intercept) 27.18329 149.97071 0.181 0.856**

**arabkm1z 0.03116 0.04598 0.678 0.498**

**arabkm3z 0.04254 0.04330 0.982 0.326**

**CALYEAR -0.01315 0.07489 0.176 0.861**

**systemO 0.11410 0.07126 1.601 0.109**

**woodkm1z 0.01908 0.04181 0.456 0.648**

**woodkm3z 0.04126 0.04627 0.892 0.373**

**arabkm1z:systemO -0.04469 0.07719 0.579 0.563**

**arabkm3z:systemO 0.01467 0.07205 0.204 0.839**

**systemO:woodkm1z -0.04983 0.07249 0.687 0.492**

**systemO:woodkm3z -0.05321 0.07172 0.742 0.458**

SPIDERS – Web builders. After harvest abundance in winter wheat cropped area 2002-2003

**Model summary:**

**Deviance AICc Delta Weight**

**2+3+4+5+8 66.91 85.05 0.00 0.10**

**2+3+4+6+8 67.07 85.21 0.16 0.10**

**2+3+4+8 69.58 85.23 0.18 0.10**

**2+3+5 72.53 85.75 0.69 0.07**

**2+3+4+6 70.19 85.83 0.78 0.07**

**2+3+4+5 70.33 85.98 0.92 0.07**

**2+3+4 72.98 86.20 1.15 0.06**

**2+3+6 73.01 86.23 1.18 0.06**

**2+3 75.71 86.57 1.52 0.05**

**2+3+4+6+10 68.54 86.69 1.63 0.05**

**2+3+4+6+8+10 66.83 87.55 2.50 0.03**

**2+3+4+5+8+9 66.89 87.62 2.56 0.03**

**3 79.41 87.98 2.92 0.02 Null model**

**2+3+4+5+9 70.14 88.29 3.23 0.02**

**3+5 77.56 88.42 3.36 0.02**

**1+3+4+5 72.88 88.53 3.47 0.02**

**1+3+4 75.59 88.81 3.75 0.02**

**1+3+5 75.68 88.90 3.84 0.02**

**3+4 78.16 89.02 3.96 0.01**

**1+3 78.53 89.38 4.33 0.01**

**3+6 78.86 89.72 4.67 0.01**

**1+3+4+6 74.22 89.87 4.82 0.01**

**3+4+5 76.71 89.93 4.87 0.01**

**1+3+6 77.39 90.61 5.56 0.01**

**1+3+4+5+7 72.54 90.68 5.63 0.01**

**1+3+4+6+10 72.56 90.71 5.65 0.01**

**3+4+6 77.75 90.97 5.92 0.01**

**1+3+4+5+9 72.85 91.00 5.94 0.01**

**1+3+4+7 75.40 91.04 5.99 0.01**

**3+4+6+10 75.58 91.23 6.17 0.00**

**3+4+5+9 76.50 92.15 7.09 0.00**

**1+3+4+6+7 74.06 92.21 7.15 0.00**

**1+3+4+5+7+9 72.53 93.26 8.21 0.00**

**1+3+4+6+7+10 72.54 93.27 8.21 0.00**

**Model-averaged coefficients:**

**Coefficient SE z value Pr(>|z|)**

**(Intercept) 0.57615 0.08798 6.549 <2e-16 *****

**arabkm1z -0.09158 0.05953 1.538 0.1239**

**arabkm3z -0.16738 0.06856 2.442 0.0146 ***

**CALYEAR2003 0.06286 0.11069 0.568 0.5701**

**systemO -0.12042 0.07541 1.597 0.1103**

**woodkm1z -0.07570 0.05002 1.513 0.1302**

**woodkm3z -0.07045 0.06391 1.102 0.2703**

**arabkm1z:systemO 0.03933 0.08804 0.447 0.6551**

**arabkm3z:systemO 0.13664 0.07519 1.817 0.0692 .**

**systemO:woodkm1z -0.01071 0.08568 0.125 0.9005**

**systemO:woodkm3z -0.08174 0.08543 0.957 0.3387**

SPIDERS – Web builders. After harvest abundance in winter wheat cropped area 2002-2003

**Organic only**

**Model summary:**

**Deviance AICc Delta Weight**

**1+3+5 4.47 38.39 0.00 0.25**

**1+3 4.85 38.80 0.42 0.20**

**1+3+4 4.53 38.90 0.52 0.19**

**3 5.37 40.17 1.78 0.10**

**2+3 5.16 41.20 2.82 0.06**

**2+3+5 4.83 41.32 2.93 0.06**

**3+4 5.19 41.40 3.01 0.05**

**2+3+4 4.87 41.65 3.26 0.05**

**3+5 5.28 42.05 3.66 0.04**

**Variables:**

**1 2 3 4 5**

**arabkm1z arabkm3z CALYEAR woodkm1z woodkm3z**

**Model-averaged coefficients:**

**Coefficient SE Adjusted SE z value Pr(>|z|)**

**(Intercept) 0.45506 0.08569 0.08879 5.125 3e-07 *****

**arabkm1z -0.13966 0.06479 0.06699 2.085 0.0371 ***

**arabkm3z -0.09824 0.06954 0.07184 1.368 0.1715**

**CALYEAR2003 0.12469 0.12148 0.12585 0.991 0.3218**

**woodkm1z -0.08908 0.06277 0.06502 1.370 0.1707**

**woodkm3z -0.10214 0.06823 0.07054 1.448 0.1476**

SPIDERS – Web builders. After harvest abundance in winter wheat cropped area 2002-2003

**conventional only**

**Model summary:**

**Deviance AICc Delta Weight**

**2+3 6.98 52.65 0.00 0.49**

**2+3+4 6.82 54.42 1.78 0.20**

**2+3+5 6.88 54.80 2.15 0.17**

**3 8.35 56.94 4.30 0.06**

**1+3 8.04 58.01 5.36 0.03**

**3+5 8.28 59.13 6.49 0.02**

**3+4 8.33 59.38 6.73 0.02**

**1+3+4 7.90 60.02 7.37 0.01**

**1+3+5 8.03 60.66 8.01 0.01**

**Variables:**

**1 2 3 4 5**

**arabkm1z arabkm3z CALYEAR woodkm1z woodkm3z**

**Model-averaged coefficients:**

**Coefficient SE Adjusted SE z value Pr(>|z|)**

**(Intercept) 0.596442 0.105025 0.108795 5.482 <2e-16 *****

**arabkm1z -0.097960 0.083466 0.086439 1.133 0.2571**

**arabkm3z -0.203492 0.077617 0.080382 2.532 0.0114 ***

**CALYEAR2003 0.007267 0.150123 0.155476 0.047 0.9627**

**woodkm1z -0.064876 0.078316 0.081126 0.800 0.4239**

**woodkm3z -0.044470 0.090506 0.093419 0.476 0.6341**

SPIDERS – Web builders. After harvest species richness in winter wheat cropped area 2002-2003

**Model summary:**

**Deviance AICc Delta Weight**

**1+3+4+5 27.29 42.93 0.00 0.07**

**2+3+5 29.75 42.97 0.03 0.07**

**2+3+6 29.85 43.07 0.13 0.06**

**2+3 32.39 43.25 0.31 0.06**

**1+3+4 30.16 43.38 0.45 0.05**

**2+3+4+6 27.74 43.39 0.45 0.05**

**2+3+4 30.33 43.55 0.61 0.05**

**2+3+4+5 28.10 43.75 0.82 0.04**

**1+3+5 30.57 43.79 0.85 0.04**

**2+3+4+6+10 25.69 43.84 0.90 0.04**

**2+3+4+8 28.19 43.84 0.91 0.04**

**1+3+4+6 28.34 43.99 1.05 0.04**

**2+3+4+6+8 25.90 44.05 1.11 0.04**

**2+3+4+5+8 26.01 44.16 1.23 0.04**

**1+3 33.56 44.42 1.48 0.03**

**1+3+4+6+10 26.31 44.46 1.53 0.03**

**1+3+4+5+9 26.71 44.86 1.93 0.03**

**2+3+4+5+9 26.88 45.02 2.09 0.02**

**3 36.49 45.05 2.12 0.02 Null model**

**1+3+4+5+7 27.13 45.28 2.34 0.02**

**1+3+6 32.08 45.30 2.36 0.02**

**1+3+4+7 30.10 45.74 2.81 0.02**

**2+3+4+6+8+10 25.10 45.83 2.89 0.02**

**3+5 35.21 46.07 3.14 0.01**

**2+3+4+5+8+9 25.46 46.19 3.26 0.01**

**1+3+4+6+7 28.30 46.45 3.51 0.01**

**3+4 35.63 46.48 3.55 0.01**

**1+3+4+6+7+10 26.14 46.87 3.93 0.01**

**3+6 36.18 47.03 4.10 0.01**

**1+3+4+5+7+9 26.69 47.42 4.48 0.01**

**3+4+5 34.63 47.84 4.91 0.01**

**3+4+6+10 32.79 48.44 5.51 0.00**

**3+4+6 35.40 48.62 5.68 0.00**

**3+4+5+9 33.38 49.03 6.09 0.00**

**Model-averaged coefficients:**

**Coefficient SE z value Pr(>|z|)**

**(Intercept) -41.15693 158.12500 0.260 0.7946**

**arabkm1z -0.09864 0.04386 2.249 0.0245 ***

**arabkm3z -0.11469 0.04736 2.422 0.0154 ***

**CALYEAR 0.02095 0.07896 0.265 0.7908**

**systemO -0.09695 0.06378 1.520 0.1285**

**woodkm1z -0.05114 0.04117 1.242 0.2141**

**woodkm3z -0.04289 0.05118 0.838 0.4020**

**arabkm1z:systemO 0.01262 0.06911 0.183 0.8551**

**arabkm3z:systemO 0.08261 0.06185 1.336 0.1817**

**systemO:woodkm1z -0.05662 0.06557 0.863 0.3879**

**systemO:woodkm3z -0.08528 0.06344 1.344 0.1789**

SPIDERS – Hunters. After harvest abundance in winter wheat field boundary 2002-2003

Model summary:

Deviance AICc Delta Weight

1+3+4+6+7 58.83 76.98 0.00 0.34

1+3+4+6+7+10 58.45 79.18 2.20 0.11

1+3+4+5+7 61.24 79.39 2.41 0.10

1+3+4+7 64.17 79.82 2.84 0.08

3+4+6 66.79 80.01 3.03 0.07

1+3+4+5+7+9 60.79 81.52 4.54 0.04

3+4+6+10 66.45 82.09 5.11 0.03

3+6 71.26 82.12 5.13 0.03

1+3+4+6 66.63 82.27 5.29 0.02

2+3+4+6 66.79 82.43 5.45 0.02

3+4+5 70.02 83.24 6.25 0.01

3+4 72.43 83.29 6.31 0.01

3+4+5+9 67.93 83.58 6.60 0.01

2+3+4+6+8 65.45 83.60 6.62 0.01

1+3+6 71.05 84.26 7.28 0.01

2+3+6 71.08 84.30 7.32 0.01

1+3+4+6+10 66.32 84.46 7.48 0.01

2+3+4 71.35 84.56 7.58 0.01

2+3+4+6+10 66.44 84.59 7.61 0.01

3+5 74.29 85.15 8.16 0.01

2+3+4+5 69.56 85.21 8.22 0.01

1+3+4 72.21 85.43 8.45 0.00

2+3+4+5+9 67.30 85.45 8.47 0.00

2+3+4+8 69.86 85.51 8.53 0.00

1+3+4+5 70.02 85.66 8.68 0.00

3 77.27 85.83 8.85 0.00 Null model

2+3 75.06 85.92 8.93 0.00

1+3+4+5+9 67.85 86.00 9.02 0.00

2+3+4+5+8 67.98 86.13 9.15 0.00

2+3+4+6+8+10 65.45 86.18 9.20 0.00

2+3+5 73.09 86.31 9.33 0.00

1+3 75.68 86.53 9.55 0.00

1+3+5 73.60 86.82 9.83 0.00

2+3+4+5+8+9 66.56 87.29 10.30 0.00

Variables:

Model-averaged coefficients:

Coefficient SE z value Pr(>|z|)

(Intercept) 0.433036 0.082872 5.225 2e-07 ***

arabkm1z 0.113468 0.072044 1.575 0.11526

arabkm3z -0.007581 0.064915 0.117 0.90703

CALYEAR2003 0.082276 0.095899 0.858 0.39092

systemO 0.164818 0.080213 2.055 0.03990 * (MORE ON ORGANIC)

woodkm1z 0.064315 0.058151 1.106 0.26872

woodkm3z 0.116900 0.054710 2.137 0.03262 *

arabkm1z:systemO -0.248311 0.085745 2.896 0.00378 **

arabkm3z:systemO -0.092271 0.081165 1.137 0.25561

systemO:woodkm1z 0.087183 0.091689 0.951 0.34168

systemO:woodkm3z -0.025455 0.094655 0.269 0.78799

SPIDERS – Hunters. After harvest abundance in winter wheat field boundary 2002-2003

Organic only

Model summary:

Deviance AICc Delta Weight

1+3+4 5.06 43.11 0.00 0.27

1+3 5.47 43.38 0.26 0.24

1+3+5 5.22 44.29 1.18 0.15

3+5 5.73 45.15 2.04 0.10

3+4 5.77 45.39 2.28 0.09

2+3+4 5.55 46.59 3.48 0.05

2+3 6.00 46.92 3.80 0.04

3 6.41 46.93 3.81 0.04

2+3+5 5.65 47.28 4.16 0.03

Model-averaged coefficients:

Coefficient SE Adjusted SE z value Pr(>|z|)

(Intercept) 0.64915 0.09121 0.09452 6.868 <2e-16 ***

arabkm1z -0.14415 0.06714 0.06950 2.074 0.0381 *

arabkm3z -0.08069 0.07355 0.07604 1.061 0.2886

CALYEAR2003 0.04467 0.12925 0.13392 0.334 0.7387

woodkm1z 0.11298 0.06635 0.06872 1.644 0.1002

woodkm3z 0.10755 0.07287 0.07529 1.428 0.1532

SPIDERS – Hunters. After harvest abundance in winter wheat field boundary 2002-2003

Conventional only

Model summary:

Deviance AICc Delta Weight

1+3+5 4.59 39.38 0.00 0.30

1+3 5.00 39.97 0.59 0.22

3 5.39 40.31 0.93 0.19

3+5 5.27 41.99 2.61 0.08

3+4 5.35 42.53 3.16 0.06

1+3+4 5.00 42.62 3.24 0.06

2+3 5.38 42.78 3.40 0.05

2+3+5 5.26 44.58 5.21 0.02

2+3+4 5.33 45.10 5.72 0.02

Variables:

1 2 3 4 5

arabkm1z arabkm3z CALYEAR woodkm1z woodkm3z

Model-averaged coefficients:

Coefficient SE Adjusted SE z value Pr(>|z|)

(Intercept) 0.438305 0.088092 0.091238 4.804 1.6e-06 ***

arabkm1z 0.127654 0.069234 0.071493 1.786 0.0742 .

arabkm3z -0.005981 0.069197 0.071609 0.084 0.9334

CALYEAR2003 0.102469 0.125889 0.130351 0.786 0.4318

woodkm1z -0.015906 0.070295 0.072629 0.219 0.8266

woodkm3z 0.101984 0.071694 0.073997 1.378 0.1681

SPIDERS – Hunters. After harvest species richness in winter wheat field boundary 2002-2003

Model summary:

Deviance AICc Delta Weight

1+3+4+6+7+10 22.85 43.58 0.00 0.24

1+3+4+6+7 25.74 43.89 0.31 0.21

3+4+6 31.30 44.52 0.95 0.15

1+3+4+6 30.42 46.06 2.49 0.07

3+4+6+10 30.89 46.54 2.96 0.05

2+3+4+6 31.27 46.92 3.34 0.05

3+6 36.13 46.99 3.41 0.04

1+3+4+6+10 29.90 48.04 4.47 0.03

1+3+4+5+7 30.77 48.92 5.35 0.02

2+3+4+6+10 30.84 48.98 5.41 0.02

2+3+4+6+8 31.03 49.18 5.61 0.01

2+3+6 36.05 49.27 5.70 0.01

1+3+6 36.13 49.34 5.77 0.01

1+3+4+7 33.84 49.49 5.91 0.01

3+4+5 36.43 49.64 6.07 0.01

3+4 38.93 49.78 6.21 0.01

2+3+4+6+8+10 29.98 50.71 7.13 0.01

2+3+4 37.62 50.84 7.26 0.01

1+3+4+5+7+9 30.66 51.38 7.81 0.00

2+3+4+5 35.82 51.46 7.89 0.00

3+5 40.91 51.77 8.19 0.00

3+4+5+9 36.32 51.97 8.40 0.00

1+3+4+5 36.35 51.99 8.42 0.00

1+3+4 38.89 52.11 8.53 0.00

2+3 41.60 52.45 8.88 0.00

3 43.95 52.52 8.94 0.00 Null model

2+3+5 39.61 52.83 9.25 0.00

2+3+4+8 37.18 52.83 9.25 0.00

2+3+4+5+8 35.42 53.57 10.00 0.00

2+3+4+5+9 35.67 53.82 10.25 0.00

1+3+5 40.67 53.89 10.32 0.00

1+3 43.04 53.90 10.32 0.00

1+3+4+5+9 36.27 54.42 10.84 0.00

2+3+4+5+8+9 35.37 56.09 12.52 0.00

Model-averaged coefficients:

Coefficient SE z value Pr(>|z|)

(Intercept) -1.448e+02 1.461e+02 0.991 0.3216

arabkm1z 1.022e-01 6.258e-02 1.633 0.1025

arabkm3z 1.182e-03 4.890e-02 0.024 0.9807

CALYEAR 7.259e-02 7.294e-02 0.995 0.3196

systemO 1.557e-01 6.663e-02 2.336 0.0195 * MORE ON ORGANIC

woodkm1z 6.187e-02 4.174e-02 1.482 0.1383

woodkm3z 1.317e-01 5.498e-02 2.396 0.0166 *

arabkm1z:systemO -1.802e-01 7.464e-02 2.415 0.0157 * INTERACTON WITH ARABLE

arabkm3z:systemO -4.385e-02 7.017e-02 0.625 0.5320

systemO:woodkm1z 2.489e-03 7.552e-02 0.033 0.9737

systemO:woodkm3z -9.882e-02 7.611e-02 1.298 0.1942

SPIDERS – Hunters. After harvest species richness in winter wheat field boundary 2002-2003

Organic only

Model summary:

Deviance AICc Delta Weight

1+3 3.22 23.30 0.00 0.21

3+5 3.27 23.83 0.54 0.16

3 3.54 24.33 1.04 0.12

1+3+4 3.10 24.49 1.20 0.11

3+4 3.34 24.64 1.34 0.11

1+3+5 3.11 24.64 1.35 0.11

2+3 3.37 25.02 1.72 0.09

2+3+5 3.24 26.10 2.80 0.05

2+3+4 3.24 26.18 2.88 0.05

Variables:

1 2 3 4 5

arabkm1z arabkm3z calyear woodkm1z woodkm3z

Model-averaged coefficients:

Coefficient SE Adjusted SE z value Pr(>|z|)

(Intercept) 0.72755 0.07043 0.07296 9.972 <2e-16 ***

arabkm1z -0.08395 0.05185 0.05368 1.564 0.118

arabkm3z -0.05453 0.05503 0.05692 0.958 0.338

calyear2003 0.05053 0.09976 0.10334 0.489 0.625

woodkm1z 0.06489 0.05146 0.05331 1.217 0.224

woodkm3z 0.07393 0.05412 0.05599 1.320 0.187

SPIDERS – Hunters. After harvest species richness in winter wheat field boundary 2002-2003

conventional only

Model summary:

Deviance AICc Delta Weight

1+3+5 2.95 22.55 0.00 0.74

3+5 3.51 26.50 3.95 0.10

3 3.91 28.14 5.59 0.05

1+3 3.72 28.77 6.22 0.03

2+3+5 3.47 28.80 6.25 0.03

1+3+4 3.58 29.98 7.43 0.02

3+4 3.88 30.34 7.79 0.01

2+3 3.89 30.48 7.93 0.01

2+3+4 3.87 32.92 10.37 0.00

Variables:

1 2 3 4 5

arabkm1z arabkm3z calyear woodkm1z woodkm3z

Model-averaged coefficients:

Coefficient SE Adjusted SE z value Pr(>|z|)

(Intercept) 0.55121 0.07025 0.07280 7.571 < 2e-16 ***

arabkm1z 0.13199 0.05520 0.05711 2.311 0.02082 *

arabkm3z 0.01448 0.06401 0.06597 0.219 0.82632

calyear2003 0.08382 0.10063 0.10426 0.804 0.42146

woodkm1z 0.04735 0.06036 0.06233 0.760 0.44752

woodkm3z 0.15291 0.05696 0.05883 2.599 0.00935 **

SPIDERS – Web builders. After harvest abundance in winter wheat field boundary 2002-2003

Model summary:

Deviance AICc Delta Weight

1+3+6 44.07 57.29 0.00 0.35

1+3 48.41 59.27 1.98 0.13

1+3+4+6 44.06 59.71 2.42 0.11

1+3+5 48.34 61.56 4.27 0.04

1+3+4 48.41 61.63 4.34 0.04

1+3+4+6+10 43.56 61.71 4.42 0.04

3 53.17 61.73 4.44 0.04 Null model

1+3+4+6+7 44.05 62.20 4.91 0.03

2+3+6 49.18 62.40 5.11 0.03

3+6 52.05 62.91 5.62 0.02

2+3 52.20 63.06 5.77 0.02

3+4 52.53 63.39 6.10 0.02

3+5 53.03 63.89 6.60 0.01

1+3+4+7 48.34 63.99 6.70 0.01

1+3+4+5 48.34 63.99 6.70 0.01

1+3+4+6+7+10 43.51 64.24 6.95 0.01

2+3+4+6 48.80 64.44 7.15 0.01

3+4+6 51.25 64.47 7.18 0.01

2+3+4 51.81 65.03 7.74 0.01

2+3+4+6+8 47.08 65.23 7.94 0.01

2+3+4+8 49.70 65.34 8.05 0.01

2+3+5 52.18 65.40 8.11 0.01

1+3+4+5+9 47.32 65.47 8.18 0.01

3+4+5 52.46 65.67 8.38 0.01

3+4+6+10 50.58 66.23 8.94 0.00

2+3+4+6+10 48.25 66.40 9.11 0.00

1+3+4+5+7 48.28 66.43 9.14 0.00

3+4+5+9 50.93 66.57 9.28 0.00

2+3+4+5 51.81 67.45 10.16 0.00

2+3+4+6+8+10 47.05 67.78 10.49 0.00

2+3+4+5+8 49.65 67.80 10.51 0.00

1+3+4+5+7+9 47.32 68.05 10.76 0.00

2+3+4+5+9 50.30 68.45 11.16 0.00

2+3+4+5+8+9 48.82 69.54 12.25 0.00

Model-averaged coefficients:

Coefficient SE z value Pr(>|z|)

(Intercept) 0.528733 0.060946 8.675 <2e-16 ***

arabkm1z -0.109974 0.045152 2.436 0.0149 *

arabkm3z -0.065654 0.051960 1.264 0.2064

CALYEAR2003 0.164546 0.079980 2.057 0.0397 *

systemO 0.007999 0.080780 0.099 0.9211

woodkm1z 0.002606 0.047467 0.055 0.9562

woodkm3z -0.082408 0.046093 1.788 0.0738 .

arabkm1z:systemO 0.004869 0.081380 0.060 0.9523

arabkm3z:systemO 0.106667 0.076181 1.400 0.1615

systemO:woodkm1z -0.084326 0.077792 1.084 0.2784

systemO:woodkm3z -0.053309 0.075805 0.703 0.4819

---

SPIDERS – Web builders. After harvest species richness in winter wheat field boundary 2002-2003

Model summary:

Deviance AICc Delta Weight

1+3+4+6 -10.21 5.44 0.00 0.32

1+3+6 -6.49 6.73 1.29 0.17

1+3+4+6+10 -10.26 7.89 2.45 0.09

1+3+4+6+7 -10.24 7.91 2.47 0.09

1+3+4 -3.85 9.36 3.92 0.05

2+3+6 -3.14 10.08 4.64 0.03

1+3 -0.76 10.10 4.66 0.03

1+3+4+6+7+10 -10.27 10.46 5.02 0.03

3 2.38 10.94 5.50 0.02

3+6 0.13 10.99 5.55 0.02

2+3+4+6 -4.30 11.35 5.91 0.02

1+3+4+5 -4.02 11.63 6.19 0.01

1+3+4+7 -4.01 11.64 6.20 0.01

1+3+5 -1.05 12.17 6.73 0.01

1+3+4+5+9 -5.84 12.31 6.87 0.01

2+3 1.58 12.43 6.99 0.01

3+4 1.68 12.53 7.09 0.01

3+4+6 -0.37 12.85 7.41 0.01

2+3+4+6+8 -5.01 13.13 7.69 0.01

3+5 2.38 13.24 7.80 0.01

2+3+4 0.45 13.66 8.22 0.01

2+3+4+6+10 -4.39 13.76 8.32 0.01

1+3+4+5+7 -4.17 13.98 8.54 0.00

3+4+5+9 -0.97 14.68 9.24 0.00

2+3+5 1.54 14.76 9.32 0.00

3+4+5 1.65 14.87 9.43 0.00

1+3+4+5+7+9 -5.84 14.89 9.45 0.00

2+3+4+8 -0.57 15.08 9.64 0.00

3+4+6+10 -0.53 15.12 9.68 0.00

2+3+4+6+8+10 -5.03 15.70 10.26 0.00

2+3+4+5+9 -2.15 16.00 10.56 0.00

2+3+4+5 0.44 16.09 10.65 0.00

2+3+4+5+8 -0.57 17.58 12.14 0.00

2+3+4+5+8+9 -2.53 18.20 12.76 0.00

Variables:

1 2 3 4 5 6 7 8 9 10

arabkm1z arabkm3z CALYEAR system woodkm1z woodkm3z arabkm1z:system arabkm3z:system system:woodkm1z system:woodkm3z

Model-averaged coefficients:

Coefficient SE z value Pr(>|z|)

(Intercept) -193.32814 118.88002 1.626 0.10390

arabkm1z -0.09232 0.03573 2.584 0.00977 **

arabkm3z -0.05683 0.03757 1.513 0.13037

CALYEAR 0.09696 0.05937 1.633 0.10243

systemO -0.09503 0.05323 1.785 0.07424 .

woodkm1z 0.00296 0.03883 0.076 0.93923

woodkm3z -0.07369 0.03306 2.229 0.02582 *

arabkm1z:systemO 0.01128 0.05400 0.209 0.83458

arabkm3z:systemO 0.04679 0.05260 0.889 0.37375

systemO:woodkm1z -0.07580 0.05377 1.410 0.15864

systemO:woodkm3z -0.01087 0.05075 0.214 0.83036

Carabid models

Carabids. Before harvest abundance in winter wheat cropped area 2002-2003

**Model summary:**

**Deviance AICc Delta Weight**

**2+3+4 234.62 247.41 0.00 0.12**

**3+4 237.19 247.75 0.34 0.10**

**2+3+4+8 233.48 248.54 1.13 0.07**

**2+3 238.00 248.56 1.15 0.07**

**2+3+4+5 233.78 248.84 1.43 0.06**

**1+3+4 236.21 249.00 1.59 0.05**

**2+3+4+6 234.50 249.55 2.15 0.04**

**3+4+5 236.85 249.64 2.23 0.04**

**3 241.36 249.73 2.32 0.04 Null model**

**1+3 239.28 249.84 2.43 0.04**

**3+4+6 237.17 249.95 2.54 0.03**

**2+3+4+5+8 232.71 250.08 2.68 0.03**

**1+3+4+7 235.05 250.11 2.70 0.03**

**2+3+5 237.62 250.40 3.00 0.03**

**1+3+4+5 235.40 250.46 3.05 0.03**

**2+3+6 237.97 250.75 3.35 0.02**

**2+3+4+6+8 233.39 250.76 3.36 0.02**

**2+3+4+5+9 233.77 251.14 3.73 0.02**

**1+3+4+6 236.21 251.27 3.86 0.02**

**2+3+4+6+10 234.02 251.39 3.98 0.02**

**1+3+5 238.79 251.58 4.17 0.01**

**1+3+4+5+7 234.23 251.60 4.19 0.01**

**3+6 241.19 251.75 4.34 0.01**

**3+4+6+10 236.80 251.85 4.44 0.01**

**3+5 241.32 251.88 4.47 0.01**

**3+4+5+9 236.84 251.90 4.49 0.01**

**1+3+6 239.27 252.06 4.65 0.01**

**1+3+4+6+7 235.04 252.41 5.00 0.01**

**2+3+4+5+8+9 232.70 252.43 5.02 0.01**

**1+3+4+5+9 235.40 252.77 5.37 0.01**

**2+3+4+6+8+10 233.27 253.00 5.59 0.01**

**1+3+4+6+10 235.67 253.04 5.63 0.01**

**1+3+4+5+7+9 234.13 253.86 6.45 0.00**

**1+3+4+6+7+10 234.83 254.56 7.16 0.00**

**Model-averaged coefficients:**

**Coefficient SE z value Pr(>|z|)**

**(Intercept) 3.2198192 0.1197326 26.892 <2e-16 *****

**arabkm1z -0.0960147 0.0785394 1.223 0.2215**

**arabkm3z -0.1336361 0.0781653 1.710 0.0873 .**

**calyear2003 0.0870793 0.1380379 0.631 0.5281**

**systemO 0.2319481 0.1201417 1.931 0.0535 (*).**

**woodkm1z -0.0520353 0.0743539 0.700 0.4840**

**woodkm3z 0.0009646 0.0754000 0.013 0.9898**

**arabkm1z:systemO 0.1381959 0.1281045 1.079 0.2807**

**arabkm3z:systemO 0.1279116 0.1222718 1.046 0.2955**

**systemO:woodkm1z -0.0013483 0.1281189 0.011 0.9916**

**systemO:woodkm3z -0.0783857 0.1281316 0.612 0.5407**

**---**

Carabids. Before harvest Species richness in winter wheat cropped area 2002-2003

**Model summary:**

**Deviance AICc Delta Weight**

**2+3 72.42 82.97 0.00 0.30**

**2+3+6 71.54 84.31 1.34 0.15**

**2+3+4 71.91 84.68 1.71 0.13**

**2+3+5 72.29 85.06 2.09 0.11**

**2+3+4+8 70.81 85.84 2.88 0.07**

**2+3+4+6 70.88 85.92 2.95 0.07**

**2+3+4+5 71.69 86.73 3.76 0.05**

**2+3+4+6+8 69.85 87.20 4.23 0.04**

**2+3+4+5+8 70.62 87.97 5.00 0.02**

**2+3+4+6+10 70.85 88.20 5.23 0.02**

**2+3+4+5+9 71.69 89.03 6.07 0.01**

**2+3+4+6+8+10 69.82 89.52 6.55 0.01**

**2+3+4+5+8+9 70.54 90.24 7.27 0.01**

**1+3 81.18 91.73 8.76 0.00**

**1+3+5 80.93 93.70 10.73 0.00**

**1+3+4 80.99 93.76 10.79 0.00**

**1+3+6 81.05 93.82 10.85 0.00**

**1+3+4+5 80.69 95.73 12.76 0.00**

**1+3+4+6 80.82 95.86 12.89 0.00**

**1+3+4+7 80.93 95.97 13.00 0.00**

**1+3+4+6+10 80.60 97.94 14.98 0.00**

**1+3+4+5+9 80.61 97.96 14.99 0.00**

**1+3+4+5+7 80.63 97.97 15.01 0.00**

**1+3+4+6+7 80.78 98.13 15.16 0.00**

**3 91.67 100.04 17.07 0.00 Null model**

**3+4 89.63 100.17 17.21 0.00**

**1+3+4+5+7+9 80.50 100.20 17.23 0.00**

**1+3+4+6+7+10 80.59 100.29 17.32 0.00**

**3+5 91.48 102.02 19.06 0.00**

**3+6 91.66 102.21 19.24 0.00**

**3+4+5 89.60 102.37 19.40 0.00**

**3+4+6 89.61 102.39 19.42 0.00**

**3+4+6+10 89.47 104.51 21.54 0.00**

**3+4+5+9 89.60 104.63 21.67 0.00**

**Model-averaged coefficients:**

**Coefficient SE z value Pr(>|z|)**

**(Intercept) 2.334738 0.050318 46.400 < 2e-16 *****

**arabkm1z -0.118176 0.035074 3.369 0.000754 *****

**arabkm3z -0.172655 0.035593 4.851 1.2e-06 *****

**calyear2003 -0.012858 0.064236 0.200 0.841352**

**systemO 0.045187 0.059006 0.766 0.443787**

**woodkm1z -0.013596 0.033873 0.401 0.688129**

**woodkm3z -0.032120 0.034267 0.937 0.348571**

**arabkm1z:systemO 0.014725 0.062903 0.234 0.814916**

**arabkm3z:systemO 0.062494 0.060061 1.041 0.298106**

**systemO:woodkm1z 0.009464 0.062329 0.152 0.879312**

**systemO:woodkm3z -0.002875 0.062971 0.046 0.963578**

**---**

Carabids. Before harvest abundance in winter wheat field boundary 2002-2003

**Model summary:**

**Deviance AICc Delta Weight**

**2+3 229.95 240.51 0.00 0.16**

**3 232.15 240.52 0.01 0.16 Null model**

**1+3 230.99 241.55 1.04 0.09**

**3+5 231.93 242.48 1.97 0.06**

**3+6 232.05 242.61 2.10 0.06**

**3+4 232.13 242.68 2.18 0.05**

**2+3+6 229.93 242.71 2.20 0.05**

**2+3+5 229.93 242.71 2.20 0.05**

**2+3+4 229.95 242.74 2.23 0.05**

**1+3+5 230.97 243.75 3.25 0.03**

**1+3+4 230.98 243.77 3.26 0.03**

**1+3+6 230.99 243.77 3.27 0.03**

**3+4+5 231.92 244.70 4.20 0.02**

**3+4+6 232.04 244.82 4.31 0.02**

**2+3+4+8 229.88 244.94 4.43 0.02**

**2+3+4+6 229.93 244.98 4.47 0.02**

**2+3+4+5 229.93 244.98 4.48 0.02**

**1+3+4+5 230.96 246.01 5.51 0.01**

**1+3+4+7 230.97 246.03 5.52 0.01**

**1+3+4+6 230.98 246.04 5.53 0.01**

**3+4+5+9 231.79 246.84 6.34 0.01**

**3+4+6+10 232.04 247.09 6.59 0.01**

**2+3+4+5+9 229.80 247.18 6.67 0.01**

**2+3+4+6+8 229.85 247.22 6.72 0.01**

**2+3+4+5+8 229.86 247.23 6.72 0.01**

**2+3+4+6+10 229.92 247.29 6.78 0.01**

**1+3+4+5+9 230.77 248.14 7.64 0.00**

**1+3+4+5+7 230.94 248.31 7.81 0.00**

**1+3+4+6+10 230.96 248.33 7.82 0.00**

**1+3+4+6+7 230.96 248.33 7.82 0.00**

**2+3+4+5+8+9 229.77 249.50 8.99 0.00**

**2+3+4+6+8+10 229.81 249.54 9.03 0.00**

**1+3+4+5+7+9 230.70 250.43 9.93 0.00**

**1+3+4+6+7+10 230.95 250.68 10.17 0.00**

**Model-averaged coefficients:**

**Coefficient SE z value Pr(>|z|)**

**(Intercept) 2.377368 0.094800 25.078 < 2e-16 *****

**arabkm1z -0.068704 0.066467 1.034 0.301**

**arabkm3z -0.093457 0.066093 1.414 0.157**

**calyear2003 0.503112 0.126159 3.988 6.67e-05 *****

**systemO 0.002277 0.126087 0.018 0.986**

**woodkm1z 0.015822 0.068378 0.231 0.817**

**woodkm3z 0.004931 0.068075 0.072 0.942**

**arabkm1z:systemO 0.017888 0.131032 0.137 0.891**

**arabkm3z:systemO -0.033425 0.126748 0.264 0.792**

**systemO:woodkm1z 0.047601 0.127657 0.373 0.709**

**systemO:woodkm3z -0.011355 0.128638 0.088 0.930**

**---**

Carabids. Before harvest species richness in winter wheat field boundary 2002-2003

Model summary:

Deviance AICc Delta Weight

2+3+4 92.76 105.53 0.00 0.19

2+3 95.48 106.03 0.50 0.15

1+3+4 94.73 107.50 1.97 0.07

2+3+4+6 92.50 107.54 2.01 0.07

2+3+4+8 92.68 107.72 2.19 0.06

2+3+4+5 92.76 107.80 2.27 0.06

2+3+6 95.09 107.86 2.33 0.06

2+3+5 95.44 108.21 2.68 0.05

1+3 98.63 109.18 3.64 0.03

2+3+4+5+9 92.37 109.71 4.18 0.02

1+3+4+5 94.68 109.72 4.19 0.02

1+3+4+7 94.69 109.73 4.20 0.02

1+3+4+6 94.72 109.76 4.23 0.02

2+3+4+6+8 92.44 109.78 4.25 0.02

2+3+4+6+10 92.50 109.84 4.31 0.02

2+3+4+5+8 92.68 110.03 4.50 0.02

1+3+5 98.50 111.27 5.74 0.01

1+3+6 98.60 111.37 5.83 0.01

1+3+4+5+9 94.56 111.91 6.37 0.01

1+3+4+5+7 94.65 111.99 6.46 0.01

1+3+4+6+10 94.66 112.01 6.48 0.01

1+3+4+6+7 94.69 112.04 6.50 0.01

2+3+4+5+8+9 92.35 112.04 6.51 0.01

2+3+4+6+8+10 92.44 112.14 6.60 0.01

1+3+4+5+7+9 94.55 114.25 8.72 0.00

1+3+4+6+7+10 94.65 114.35 8.82 0.00

3 106.15 114.51 8.98 0.00 Null model

3+4 104.55 115.10 9.57 0.00

3+5 105.88 116.43 10.90 0.00

3+6 106.00 116.55 11.02 0.00

3+4+5 104.02 116.79 11.26 0.00

3+4+6 104.28 117.05 11.52 0.00

3+4+5+9 103.68 118.71 13.18 0.00

3+4+6+10 104.27 119.30 13.77 0.00

Model-averaged coefficients:

Coefficient SE z value Pr(>|z|)

(Intercept) 1.938e+00 6.216e-02 31.176 < 2e-16 ***

arabkm1z -1.138e-01 3.856e-02 2.952 0.003157 **

arabkm3z -1.241e-01 3.723e-02 3.333 0.000858 ***

calyear2003 2.500e-01 6.790e-02 3.681 0.000232 ***

systemO -1.182e-01 6.915e-02 1.709 0.087413 (*)

woodkm1z -6.606e-05 3.990e-02 0.002 0.998679

woodkm3z -1.532e-02 3.858e-02 0.397 0.691274

arabkm1z:systemO 1.253e-02 7.115e-02 0.176 0.860171

arabkm3z:systemO 1.797e-02 6.838e-02 0.263 0.792677

systemO:woodkm1z -3.763e-02 6.924e-02 0.543 0.586820

systemO:woodkm3z -6.629e-03 6.972e-02 0.095 0.924249

---

Carabids. After harvest abundance in winter wheat cropped area 2002-2003

**Model summary:**

**Deviance AICc Delta Weight**

**3 146.89 155.45 0.00 0.16 Null model**

**2+3 145.92 156.78 1.33 0.08**

**3+4 146.27 157.13 1.68 0.07**

**3+6 146.36 157.22 1.76 0.07**

**2+3+6 144.08 157.30 1.84 0.07**

**1+3 146.69 157.54 2.09 0.06**

**3+5 146.70 157.56 2.10 0.06**

**2+3+5 145.43 158.65 3.19 0.03**

**2+3+4 145.54 158.76 3.31 0.03**

**2+3+4+8 143.20 158.85 3.40 0.03**

**3+4+6 145.64 158.86 3.40 0.03**

**1+3+6 145.69 158.91 3.46 0.03**

**3+4+5+9 143.33 158.98 3.52 0.03**

**2+3+4+5+8+9 138.41 159.14 3.69 0.03**

**3+4+5 145.98 159.20 3.74 0.03**

**2+3+4+6+8 141.15 159.30 3.85 0.02**

**2+3+4+6 143.70 159.35 3.90 0.02**

**1+3+4 146.24 159.46 4.01 0.02**

**1+3+5 146.30 159.52 4.07 0.02**

**2+3+4+5+9 142.30 160.45 5.00 0.01**

**2+3+4+5 144.96 160.61 5.16 0.01**

**2+3+4+5+8 142.64 160.79 5.34 0.01**

**1+3+4+6 145.34 160.99 5.53 0.01**

**3+4+6+10 145.49 161.14 5.69 0.01**

**1+3+4+7 145.66 161.31 5.85 0.01**

**1+3+4+5+9 143.29 161.44 5.99 0.01**

**1+3+4+5 145.85 161.49 6.04 0.01**

**2+3+4+6+10 143.52 161.67 6.22 0.01**

**2+3+4+6+8+10 141.04 161.77 6.31 0.01**

**1+3+4+5+7+9 141.80 162.52 7.07 0.00**

**1+3+4+6+7 144.73 162.88 7.43 0.00**

**1+3+4+6+10 145.18 163.33 7.88 0.00**

**1+3+4+5+7 145.36 163.51 8.05 0.00**

**1+3+4+6+7+10 144.72 165.44 9.99 0.00**

**Model-averaged coefficients:**

**Coefficient SE z value Pr(>|z|)**

**(Intercept) 2.147920 0.116956 18.365 <2e-16 *****

**arabkm1z 0.029413 0.087950 0.334 0.7381**

**arabkm3z 0.060209 0.103323 0.583 0.5601**

**calyear2003 0.198295 0.147632 1.343 0.1792**

**systemO -0.103077 0.144911 0.711 0.4769**

**woodkm1z 0.003273 0.105338 0.031 0.9752**

**woodkm3z 0.088235 0.087703 1.006 0.3144**

**arabkm1z:systemO 0.135150 0.160158 0.844 0.3988**

**arabkm3z:systemO 0.244338 0.147058 1.662 0.0966 .**

**systemO:woodkm1z 0.263755 0.148703 1.774 0.0761 .**

**systemO:woodkm3z -0.029996 0.157431 0.191 0.8489**

Carabids. After harvest species richness in winter wheat cropped area 2002-2003

**Model summary:**

**Deviance AICc Delta Weight**

**3 55.36 63.92 0.00 0.20 Null model**

**3+4 54.43 65.28 1.36 0.10**

**2+3 54.72 65.58 1.65 0.09**

**3+6 55.10 65.96 2.03 0.07**

**1+3 55.24 66.10 2.17 0.07**

**3+5 55.36 66.22 2.29 0.06**

**2+3+6 53.78 67.00 3.07 0.04**

**3+4+6 54.06 67.27 3.35 0.04**

**2+3+4 54.06 67.27 3.35 0.04**

**3+4+5 54.41 67.62 3.70 0.03**

**1+3+4 54.42 67.64 3.72 0.03**

**2+3+5 54.69 67.91 3.98 0.03**

**1+3+6 54.76 67.98 4.06 0.03**

**3+4+5+9 52.76 68.41 4.48 0.02**

**1+3+5 55.23 68.45 4.52 0.02**

**2+3+4+8 52.90 68.55 4.63 0.02**

**2+3+4+6 53.11 68.76 4.84 0.02**

**2+3+4+5 53.99 69.64 5.71 0.01**

**1+3+4+6 54.02 69.67 5.75 0.01**

**3+4+6+10 54.05 69.70 5.78 0.01**

**1+3+4+7 54.31 69.96 6.04 0.01**

**2+3+4+6+8 51.82 69.97 6.05 0.01**

**1+3+4+5 54.41 70.05 6.13 0.01**

**2+3+4+5+9 52.37 70.52 6.59 0.01**

**1+3+4+5+9 52.73 70.88 6.96 0.01**

**2+3+4+5+8 52.83 70.98 7.06 0.01**

**2+3+4+5+8+9 50.28 71.01 7.08 0.01**

**2+3+4+6+10 53.10 71.25 7.33 0.01**

**1+3+4+6+7 53.90 72.05 8.13 0.00**

**1+3+4+6+10 54.02 72.17 8.24 0.00**

**2+3+4+6+8+10 51.57 72.30 8.38 0.00**

**1+3+4+5+7 54.30 72.45 8.53 0.00**

**1+3+4+5+7+9 52.17 72.90 8.98 0.00**

**1+3+4+6+7+10 53.89 74.62 10.70 0.00**

**Model-averaged coefficients:**

**Coefficient SE z value Pr(>|z|)**

**(Intercept) 1.7448004 0.0689603 25.302 <2e-16 *****

**arabkm1z 0.0085254 0.0470557 0.181 0.856**

**arabkm3z 0.0315407 0.0509476 0.619 0.536**

**calyear2003 0.1147990 0.0894784 1.283 0.199**

**systemO -0.0666609 0.0726542 0.918 0.359**

**woodkm1z -0.0060789 0.0498356 0.122 0.903**

**woodkm3z 0.0321866 0.0477907 0.673 0.501**

**arabkm1z:systemO 0.0316336 0.0816401 0.387 0.698**

**arabkm3z:systemO 0.0857336 0.0741371 1.156 0.248**

**systemO:woodkm1z 0.1056001 0.0780824 1.352 0.176**

**systemO:woodkm3z 0.0004423 0.0780682 0.006 0.995**

Carabids. After harvest abundance in winter wheat field boundary 2002-2003

**Model summary:**

**Deviance AICc Delta Weight**

**1+3+4 132.16 145.37 0.00 0.11**

**3+4 134.76 145.62 0.24 0.10**

**1+3+4+6 130.28 145.93 0.56 0.08**

**3 137.41 145.98 0.60 0.08 Null model**

**1+3+4+7 131.12 146.77 1.40 0.05**

**1+3+4+6+7 129.01 147.16 1.79 0.04**

**2+3+4 134.01 147.23 1.86 0.04**

**1+3+4+5 131.78 147.43 2.05 0.04**

**3+4+6 134.28 147.50 2.13 0.04**

**3+6 136.68 147.54 2.16 0.04**

**1+3 136.78 147.63 2.26 0.04**

**3+4+5 134.73 147.94 2.57 0.03**

**2+3 137.16 148.02 2.64 0.03**

**3+5 137.18 148.04 2.67 0.03**

**1+3+4+6+10 129.96 148.11 2.74 0.03**

**2+3+4+6 132.50 148.15 2.78 0.03**

**1+3+6 135.17 148.39 3.02 0.02**

**2+3+6 135.59 148.81 3.44 0.02**

**1+3+4+5+7 130.78 148.93 3.56 0.02**

**2+3+4+8 133.54 149.18 3.81 0.02**

**1+3+5 136.15 149.36 3.99 0.01**

**2+3+4+5 133.86 149.51 4.13 0.01**

**1+3+4+6+7+10 129.00 149.73 4.35 0.01**

**3+4+6+10 134.11 149.76 4.39 0.01**

**1+3+4+5+9 131.74 149.89 4.52 0.01**

**2+3+5 136.74 149.96 4.58 0.01**

**2+3+4+6+8 131.90 150.05 4.68 0.01**

**3+4+5+9 134.72 150.37 5.00 0.01**

**2+3+4+6+10 132.27 150.42 5.04 0.01**

**1+3+4+5+7+9 130.77 151.50 6.12 0.01**

**2+3+4+5+8 133.37 151.52 6.15 0.01**

**2+3+4+5+9 133.86 152.01 6.63 0.00**

**2+3+4+6+8+10 131.89 152.62 7.24 0.00**

**2+3+4+5+8+9 133.33 154.06 8.68 0.00**

**Model-averaged coefficients:**

**Coefficient SE z value Pr(>|z|)**

**(Intercept) 1.465764 0.129757 11.296 <2e-16 *****

**arabkm1z -0.110738 0.090879 1.219 0.2230**

**arabkm3z -0.066889 0.085580 0.782 0.4345**

**calyear2003 0.342129 0.148416 2.305 0.0212 ***

**systemO -0.253562 0.132904 1.908 0.0564 (*)**

**woodkm1z -0.036897 0.077839 0.474 0.6355**

**woodkm3z -0.097779 0.085559 1.143 0.2531**

**arabkm1z:systemO -0.143826 0.135553 1.061 0.2887**

**arabkm3z:systemO -0.091170 0.128306 0.711 0.4774**

**systemO:woodkm1z 0.003375 0.133712 0.025 0.9799**

**systemO:woodkm3z 0.053146 0.131297 0.405 0.6856**

Carabids. After harvest species richness in winter wheat field boundary 2002-2003

Model summary:

Deviance AICc Delta Weight

3+4 73.96 84.82 0.00 0.19

1+3+4 73.31 86.52 1.71 0.08

3+4+6 73.61 86.82 2.01 0.07

3 78.27 86.83 2.02 0.07 Null model

2+3+4 73.87 87.09 2.27 0.06

3+4+5 73.94 87.16 2.34 0.06

1+3+4+7 72.16 87.81 2.99 0.04

1+3+4+6 72.41 88.06 3.24 0.04

3+6 77.58 88.44 3.62 0.03

3+4+6+10 72.82 88.47 3.65 0.03

2+3+4+8 72.86 88.51 3.69 0.03

2+3+4+6 73.21 88.85 4.03 0.03

1+3+4+5 73.30 88.95 4.13 0.02

3+5 78.17 89.02 4.21 0.02

2+3 78.25 89.11 4.29 0.02

1+3 78.26 89.12 4.30 0.02

1+3+4+6+7 71.12 89.27 4.46 0.02

2+3+4+5 73.87 89.51 4.70 0.02

3+4+5+9 73.91 89.55 4.73 0.02

1+3+4+6+10 71.46 89.61 4.79 0.02

2+3+4+6+8 72.09 90.24 5.43 0.01

1+3+4+5+7 72.16 90.31 5.49 0.01

2+3+4+6+10 72.36 90.51 5.69 0.01

2+3+6 77.50 90.72 5.90 0.01

1+3+6 77.53 90.75 5.93 0.01

2+3+4+5+8 72.85 91.00 6.18 0.01

1+3+4+5+9 73.20 91.35 6.53 0.01

2+3+5 78.16 91.38 6.56 0.01

1+3+5 78.17 91.38 6.57 0.01

1+3+4+6+7+10 70.82 91.55 6.73 0.01

2+3+4+5+9 73.83 91.98 7.16 0.01

2+3+4+6+8+10 71.88 92.61 7.79 0.00

1+3+4+5+7+9 72.16 92.89 8.07 0.00

2+3+4+5+8+9 72.84 93.57 8.75 0.00

Model-averaged coefficients:

Coefficient SE z value Pr(>|z|)

(Intercept) 1.455359 0.088881 16.374 <2e-16 ***

arabkm1z -0.028857 0.061420 0.470 0.6385

arabkm3z -0.007667 0.059857 0.128 0.8981

calyear2003 0.201874 0.104782 1.927 0.0540

systemO -0.176795 0.082040 2.155 0.0312 *

woodkm1z -0.001629 0.052321 0.031 0.9752

woodkm3z -0.051429 0.059727 0.861 0.3892

arabkm1z:systemO -0.095472 0.089658 1.065 0.2869

arabkm3z:systemO -0.081839 0.082206 0.996 0.3195

systemO:woodkm1z 0.015244 0.087686 0.174 0.8620

systemO:woodkm3z 0.072150 0.085139 0.847 0.3968

System+Habitat effects

Predictor Variables for cropped area models:

1. arabkm3z Arable extent (%) in 9km^2^ in landscape
2. CALYEAR Calendar year
3. MEAN_SCT Mean plant species diversity in cropped area
4. smeanhb Mean field hedgerow bulk (sqrt)
5. system Organic or Conventional

Interaction effects

1. arabkm3z:system
2. MEAN_SCT:system
3. smeanhb:system

Predictor Variables for field boundary models:

1 arabkm1z Arable extent (%) in 1 km^2^ in landscape

2 CALYEAR Calendar year

3 MEAN_MW Mean margin width

4 smeanhb Mean field hedgerow bulk (sqrt)

5 SP_CODE_TOT_B Mean plant species diversity in boundary plots

6 system Organic conventional

7 arabkm1z:system

8 MEAN_MW:system

9 smeanhb:system

10 SP_CODE_TOT_B:system

Hunting spiders cropped area ww before harvest abundance

**Model summary:**

**Deviance AICc Delta Weight**

**1+2+3+5+6+7 198.45 218.18 0.00 0.25**

**1+2+3+4+5+6+7 196.08 218.21 0.03 0.24**

**1+2+4+5+6 202.46 219.84 1.65 0.11**

**1+2+3+4+5+6 200.13 219.86 1.68 0.11**

**1+2+3+4+5+6+7+8 195.39 219.98 1.80 0.10**

**1+2+5+6 206.13 221.18 3.00 0.06**

**1+2+3+5+6 203.97 221.34 3.16 0.05**

**1+2+4+5+6+8 201.68 221.41 3.23 0.05**

**1+2+3+4+5+6+8 199.75 221.88 3.70 0.04**

**Model-averaged coefficients:**

**Coefficient SE z value Pr(>|z|)**

**(Intercept) 0.96944 0.32096 3.020 0.00252 ****

**arabkm3z 0.23354 0.07653 3.052 0.00228 ****

**as.factor(CALYEAR)2003 -0.35784 0.13917 2.571 0.01013 ***

**MEAN_SCT 0.09133 0.05499 1.661 0.09675 .**

**smeanhb 0.02767 0.01639 1.688 0.09137 .**

**systemO 0.82432 0.36429 2.263 0.02365 ***

**arabkm3z:systemO -0.30444 0.09550 3.188 0.00143 ****

**MEAN_SCT:systemO -0.10077 0.04501 2.239 0.02517 ***

**smeanhb:systemO -0.02565 0.03182 0.806 0.42024**

Hunting spiders cropped area ww before harvest abundance

Organic models

**Model summary:**

**Deviance AICc Delta Weight**

**1+2 24.21 121.73 0.00 0.45**

**1+2+3 23.74 123.00 1.27 0.24**

**1+2+4 23.94 123.50 1.77 0.19**

**1+2+3+4 23.29 124.43 2.71 0.12**

**Variables:**

**1 2 3 4**

**arabkm3z as.factor(CALYEAR) MEAN_SCT smeanhb**

**Model-averaged coefficients:**

**Coefficient SE Adjusted SE z value Pr(>|z|)**

**(Intercept) 1.89838 0.24478 0.24866 7.635 <2e-16 *****

**arabkm3z -0.03172 0.09164 0.09373 0.338 0.7350**

**as.factor(CALYEAR)2003 -0.38906 0.17897 0.18310 2.125 0.0336 ***

**MEAN_SCT 0.02107 0.01941 0.01987 1.060 0.2889**

**smeanhb 0.02526 0.02967 0.03036 0.832 0.4054**

Hunting spiders cropped area ww before harvest abundance

Conventional Models

**Model summary:**

**Deviance AICc Delta Weight**

**1+2+3+4 18.02 109.79 0.00 0.38**

**1+2+3 18.83 109.82 0.02 0.37**

**1+2 20.19 111.36 1.57 0.17**

**1+2+4 19.90 112.94 3.15 0.08**

**Variables:**

**1 2 3 4**

**arabkm3z as.factor(CALYEAR) MEAN_SCT smeanhb**

**Model-averaged coefficients:**

**Coefficient SE Adjusted SE z value Pr(>|z|)**

**(Intercept) 0.96825 0.36599 0.37012 2.616 0.00890 ****

**arabkm3z 0.24749 0.08191 0.08380 2.953 0.00314 ****

**as.factor(CALYEAR)2003 -0.35940 0.16448 0.16821 2.137 0.03263 ***

**MEAN_SCT 0.10307 0.04924 0.05035 2.047 0.04066 ***

**smeanhb 0.03867 0.02800 0.02863 1.351 0.17677**

Hunting spiders cropped area ww before harvest species richness

**Model summary:**

**Deviance AICc Delta Weight**

**1+2+3+5+6+7 58.90 78.63 0.00 0.23**

**1+2+3+4+5+6+7 57.06 79.20 0.57 0.17**

**1+2+4+5+6 62.25 79.62 0.99 0.14**

**1+2+3+4+5+6+7+8 55.12 79.71 1.08 0.13**

**1+2+4+5+6+8 60.60 80.33 1.70 0.10**

**1+2+5+6 65.43 80.48 1.85 0.09**

**1+2+3+4+5+6 61.64 81.38 2.74 0.06**

**1+2+3+5+6 64.90 82.27 3.64 0.04**

**1+2+3+4+5+6+8 60.29 82.42 3.79 0.03**

**Model-averaged coefficients:**

**Coefficient SE z value Pr(>|z|)**

**(Intercept) 1.078087 0.175675 6.137 < 2e-16 *****

**arabkm3z 0.066698 0.041440 1.609 0.107508**

**as.factor(CALYEAR)2003 -0.243497 0.075347 3.232 0.001231 ****

**MEAN_SCT 0.048188 0.029855 1.614 0.106510**

**smeanhb 0.015905 0.009764 1.629 0.103330**

**systemO 0.501943 0.211665 2.371 0.017721 ***

**arabkm3z:systemO -0.187135 0.051379 3.642 0.000270 *****

**MEAN_SCT:systemO -0.058379 0.024292 2.403 0.016253 ***

**smeanhb:systemO -0.022887 0.017146 1.335 0.181929**

**Hunting spiders cropped area ww before harvest abundance species richness**

**Organic models**

**Model summary:**

**Deviance AICc Delta Weight**

**1+2 6.75 48.94 0.00 0.57**

**1+2+3 6.73 51.15 2.21 0.19**

**1+2+4 6.74 51.20 2.26 0.18**

**1+2+3+4 6.70 53.42 4.48 0.06**

**Variables:**

**1 2 3 4**

**arabkm3z as.factor(CALYEAR) MEAN_SCT smeanhb**

**Model-averaged coefficients:**

**Coefficient SE Adjusted SE z value Pr(>|z|)**

**(Intercept) 1.603344 0.100229 0.102391 15.659 <2e-16 *****

**arabkm3z -0.098317 0.048030 0.049139 2.001 0.0454 ***

**as.factor(CALYEAR)2003 -0.256040 0.094515 0.096700 2.648 0.0081 ****

**MEAN_SCT 0.004630 0.010305 0.010547 0.439 0.6606**

**smeanhb 0.006169 0.015661 0.016028 0.385 0.7003**

**Hunting spiders cropped area ww before harvest abundance species richness**

**Conventional models**

Component models:

df logLik AICc Delta Weight

1+2+3 5 -15.80 42.78 0.00 0.39

1+2+3+4 6 -14.96 43.59 0.81 0.26

1+2 4 -17.44 43.65 0.87 0.25

1+2+44 5 -17.19 45.57 2.79 0.10

Term codes:

arabkm3z as.factor(CALYEAR) MEAN_SCT smeanhb

1 2 3 4

Model-averaged coefficients:

Estimate Std. Error Adjusted SE z value Pr(>|z|)

(Intercept) 1.09956 0.18841 0.19057 5.770 1e-08

MEAN_SCT 0.05047 0.02713 0.02775 1.819 0.06891

arabkm3z 0.06502 0.04543 0.04648 1.399 0.16185

as.factor(CALYEAR)2003 -0.24798 0.09137 0.09344 2.654 0.00796

smeanhb 0.01662 0.01566 0.01601 1.038 0.29920

Carabids cropped area ww before harvest abundance

Model summary:

df logLik AICc Delta Weight

123567 9 -29.45 78.63 0.00 0.23

1234567 10 -28.53 79.20 0.57 0.17

12456 8 -31.13 79.62 0.99 0.14

12345678 11 -27.56 79.71 1.08 0.13

124568 9 -30.30 80.33 1.70 0.10

1256 7 -32.71 80.48 1.85 0.09

123456 9 -30.82 81.38 2.74 0.06

12356 8 -32.45 82.27 3.64 0.04

1234568 10 -30.14 82.42 3.79 0.03

Model-averaged coefficients:

Estimate Std. Error Adjusted SE z value Pr(>|z|)

(Intercept) 0.57614 0.37048 0.37194 1.549 0.121377

MEAN_SCT 0.09518 0.06103 0.06137 1.551 0.120924

arabkm3z 0.25383 0.08170 0.08264 3.072 0.002129

as.factor(CALYEAR)2003 -0.24350 0.07535 0.07621 3.195 0.001398

system 0.50195 0.21167 0.21264 2.361 0.018250

MEAN_SCT:system -0.05838 0.02429 0.02457 2.376 0.017516 *

arabkm3z:system -0.18713 0.05138 0.05197 3.601 0.000318 **

smeanhb 0.02548 0.02295 0.02310 1.103 0.269989

smeanhb:system -0.02289 0.01715 0.01735 1.319 0.187046

Organic models

Component models:

df logLik AICc Delta Weight

123 5 -55.27 121.71 0.00 0.53

12 4 -57.28 123.33 1.62 0.24

1234 6 -55.26 124.19 2.48 0.15

124 5 -57.14 125.46 3.75 0.08

Term codes:

arabkm3z as.factor(CALYEAR) MEAN_SCT smeanhb

1 2 3 4

Model-averaged coefficients:

Estimate Std. Error Adjusted SE z value Pr(>|z|)

(Intercept) 3.736620 0.326768 0.330946 11.291 <2e-16

MEAN_SCT -0.037305 0.019122 0.019570 1.906 0.0566

arabkm3z -0.065697 0.093857 0.095942 0.685 0.4935

as.factor(CALYEAR)2003 0.122509 0.179809 0.183946 0.666 0.5054

smeanhb 0.008219 0.030169 0.030862 0.266 0.7900

Conventional Models

df logLik AICc Delta Weight

12 4 -59.90 128.56 0.00 0.58

124 5 -59.79 130.76 2.19 0.19

123 5 -59.89 130.95 2.38 0.17

1234 6 -59.75 133.17 4.61 0.06

Term codes:

arabkm3z as.factor(CALYEAR) MEAN_SCT smeanhb

1 2 3 4

Model-averaged coefficients:

Estimate Std. Error Adjusted SE z value Pr(>|z|)

(Intercept) 3.15076 0.20545 0.21009 14.997 <2e-16

arabkm3z -0.18725 0.09671 0.09895 1.892 0.0584

as.factor(CALYEAR)2003 0.09938 0.19146 0.19589 0.507 0.6119

smeanhb 0.01484 0.03214 0.03290 0.451 0.6519

MEAN_SCT 0.01064 0.05788 0.05923 0.180 0.8574

Post Harvest boundary hunting spider abundance

| Component models:  df logLik AICc Delta Weight  1+2+6+7 7 -31.55 78.74 0.00 0.19  1+2+3+6+7 8 -30.35 78.85 0.10 0.18  1+2+4+6+7 8 -30.67 79.48 0.74 0.13  1+2+3+4+6+7 9 -29.80 80.32 1.58 0.08  1+2+5+6+7 8 -31.35 80.85 2.11 0.06  1+2+3+5+6+7 9 -30.25 81.23 2.48 0.05  1+2+3+6+7+8 9 -30.32 81.36 2.62 0.05  1+2+4+6+7+9 9 -30.59 81.90 3.16 0.04  1+2+4+5+6+7 9 -30.59 81.91 3.17 0.04  1+2+3+4+6+7+8 10 -29.69 82.77 4.03 0.02  1+2+3+4+5+6+7 10 -29.76 82.90 4.15 0.02  1+2+3+4+6+7+9 10 -29.77 82.93 4.19 0.02  1+2+5+6+7+10 9 -31.28 83.29 4.55 0.02  1+2+3+5+6+7+10 10 -30.20 83.79 5.04 0.01  1+2+3+5+6+7+8 10 -30.23 83.85 5.11 0.01  1+2+4+5+6+7+9 10 -30.53 84.44 5.70 0.01  1+2+4+5+6+7+10 10 -30.59 84.57 5.82 0.01  1+2+3+4+6+7+8+9 11 -29.65 85.43 6.69 0.01  1+2+3+4+5+6+7+8 11 -29.67 85.47 6.72 0.01  1+2+3+4+5+6+7+9 11 -29.74 85.60 6.86 0.01  1+2+3+4+5+6+7+10 11 -29.75 85.63 6.89 0.01  1+2+3+5+6+7+8+10 11 -30.17 86.47 7.73 0.00  1+2+4+5+6+7+9+10 11 -30.53 87.18 8.44 0.00  1+2+3+4+5+6+7+8+9 12 -29.64 88.23 9.49 0.00  1+2+3+4+5+6+7+8+10 12 -29.67 88.29 9.54 0.00  1+2+3+4+5+6+7+9+10 12 -29.74 88.43 9.68 0.00  1+2+3+4+5+6+7+8+9+10 13 -29.64 91.14 12.40 0.00  Model-averaged coefficients:  Estimate Std. Error Adjusted SE z value Pr(>\|z\|)  (Intercept) 0.342499 0.126857 0.128755 2.660 0.00781  arabkm1z 0.089880 0.061196 0.062307 1.443 0.14915  as.factor(CALYEAR)2003 0.046582 0.095241 0.096940 0.481 0.63085  systemO 0.154172 0.118073 0.120153 1.283 0.19945  arabkm1z:systemO -0.248724 0.085430 0.086983 2.859 0.00424 **  MEAN_MW 0.042553 0.032458 0.033043 1.288 0.19781  smeanhb 0.014273 0.011932 0.012146 1.175 0.23996  SP_CODE_TOT_B 0.004840 0.010147 0.010328 0.469 0.63935  MEAN_MW:systemO 0.018480 0.057821 0.058885 0.314 0.75366  smeanhb:systemO -0.008057 0.024511 0.024964 0.323 0.74689  SP_CODE_TOT_B:systemO -0.003877 0.016273 0.016570 0.234 0.81501    Organic Models  Component models:  df logLik AICc Delta Weight  1+2 4 -16.87 42.95 0.00 0.37  1+2+3 5 -16.10 44.07 1.12 0.21  1+2+4 5 -16.68 45.23 2.29 0.12  1+2+5 5 -16.72 45.31 2.37 0.11  1+2+3+4 6 -15.74 46.19 3.25 0.07  1+2+3+5 6 -15.94 46.59 3.64 0.06  1+2+4+5 6 -16.54 47.79 4.84 0.03  1+2+3+4+5 7 -15.60 48.93 5.98 0.02  Model-averaged coefficients:  Estimate Std. Error Adjusted SE z value Pr(>\|z\|)  (Intercept) 0.56212 0.18568 0.19065 2.948 0.00319  arabkm1z -0.15757 0.06675 0.06916 2.278 0.02270 *  as.factor(CALYEAR)2003 0.01836 0.13097 0.13570 0.135 0.89236  MEAN_MW 0.06184 0.05118 0.05309 1.165 0.24405  MEAN_SCT 0.01087 0.01665 0.01727 0.629 0.52918  smeanhb 0.01129 0.02201 0.02284 0.494 0.62107  Conventional Models  df logLik AICc Delta Weight  1+2 4 -15.05 39.32 0.00 0.42  1+2+3 5 -14.54 40.95 1.64 0.19  1+2+5 5 -14.96 41.80 2.48 0.12  1+2+4 5 -14.98 41.83 2.51 0.12  1+2+3+5 6 -14.42 43.55 4.24 0.05  1+2+3+4 6 -14.45 43.60 4.28 0.05  1+2+4+5 6 -14.91 44.54 5.22 0.03  1+2+3+4+5 7 -14.36 46.46 7.14 0.01  Term codes:  arabkm1z as.factor(CALYEAR) MEAN_MW MEAN_SCT smeanhb  1 2 3 4 5  Model-averaged coefficients:  Estimate Std. Error Adjusted SE z value Pr(>\|z\|)  (Intercept) 0.436365 0.148242 0.153073 2.851 0.00436  arabkm1z 0.096632 0.062828 0.065125 1.484 0.13786  as.factor(CALYEAR)2003 0.072878 0.127189 0.131808 0.553 0.58032  MEAN_MW 0.038176 0.039447 0.040921 0.933 0.35086  smeanhb 0.008565 0.021333 0.022131 0.387 0.69874  MEAN_SCT -0.015188 0.042186 0.043764 0.347 0.72856 |
| --- |
|  |
| \|  \| \| --- \| |

Post Harvest boundary carabid abundance

| Component models:  df logLik AICc Delta Weight  1+2+3+6+7+8 9 -61.94 144.60 0.00 0.13  1+2+4+5+6+7 9 -61.96 144.64 0.04 0.12  1+2+3+4+6+7+8 10 -61.00 145.38 0.78 0.08  1+2+3+4+5+6+7+8 11 -59.68 145.48 0.88 0.08  1+2+4+6+7 8 -63.83 145.81 1.21 0.07  1+2+3+5+6+7+8 10 -61.28 145.94 1.34 0.06  1+2+3+4+6+7+8+9 11 -60.27 146.66 2.06 0.04  1+2+6+7 7 -65.56 146.77 2.17 0.04  1+2+4+5+6+7+10 10 -61.72 146.82 2.23 0.04  1+2+4+5+6+7+9 10 -61.86 147.10 2.50 0.04  1+2+3+4+5+6+7 10 -61.92 147.23 2.63 0.03  1+2+5+6+7 8 -64.66 147.47 2.87 0.03  1+2+3+4+5+6+7+8+9 12 -59.30 147.55 2.96 0.03  1+2+4+6+7+9 9 -63.49 147.72 3.12 0.03  1+2+3+5+6+7+8+10 11 -60.84 147.80 3.20 0.03  1+2+3+4+5+6+7+8+10 12 -59.57 148.09 3.49 0.02  1+2+5+6+7+10 9 -63.76 148.24 3.64 0.02  1+2+3+4+6+7 9 -63.82 148.37 3.77 0.02  1+2+3+6+7 8 -65.46 149.07 4.47 0.01  1+2+4+5+6+7+9+10 11 -61.52 149.17 4.57 0.01  1+2+3+4+5+6+7+10 11 -61.68 149.48 4.89 0.01  1+2+3+5+6+7 9 -64.49 149.70 5.10 0.01  1+2+3+4+5+6+7+9 11 -61.81 149.74 5.14 0.01  1+2+3+4+5+6+7+8+9+10 13 -59.07 150.01 5.41 0.01  1+2+3+4+6+7+9 10 -63.47 150.33 5.73 0.01  1+2+3+5+6+7+10 10 -63.61 150.60 6.01 0.01  1+2+3+4+5+6+7+9+10 12 -61.46 151.88 7.28 0.00  Model-averaged coefficients:  Estimate Std. Error Adjusted SE z value Pr(>\|z\|)  (Intercept) 1.58900 0.26533 0.26838 5.921 <2e-16  MEAN_MW -0.08398 0.06501 0.06589 1.275 0.2024  arabkm1z -0.05200 0.09369 0.09539 0.545 0.5857  as.factor(CALYEAR)2003 0.28303 0.15549 0.15804 1.791 0.0733  systemO -0.64349 0.37553 0.37861 1.700 0.0892(*)  MEAN_MW:systemO 0.21273 0.08812 0.08971 2.371 0.0177 *  arabkm1z:systemO -0.15083 0.13280 0.13514 1.116 0.2644  smeanhb -0.03053 0.02076 0.02107 1.449 0.1473  SP_CODE_TOT_B 0.01920 0.01743 0.01769 1.085 0.2779  smeanhb:systemO -0.03091 0.03862 0.03930 0.787 0.4316  SP_CODE_TOT_B:systemO 0.02050 0.02560 0.02605 0.787 0.4313  Organic models  Component models:  df logLik AICc Delta Weight  12 4 -37.60 84.41 0.00 0.19  125 5 -36.39 84.65 0.24 0.17  124 5 -36.46 84.79 0.39 0.16  1245 6 -35.19 85.10 0.69 0.14  123 5 -36.77 85.41 1.00 0.12  1234 6 -35.58 85.88 1.47 0.09  1235 6 -35.81 86.34 1.93 0.07  12345 7 -34.59 86.91 2.50 0.06  Term codes:  arabkm1z as.factor(CALYEAR) MEAN_MW smeanhb SP_CODE_TOT_B  1 2 3 4 5  Model-averaged coefficients:  Estimate Std. Error Adjusted SE z value Pr(>\|z\|)  (Intercept) 1.04532 0.37825 0.38656 2.704 0.00685  arabkm1z -0.18284 0.11223 0.11630 1.572 0.11592  as.factor(CALYEAR)2003 0.30369 0.25812 0.26624 1.141 0.25402  SP_CODE_TOT_B 0.03270 0.02273 0.02358 1.387 0.16547  smeanhb -0.05333 0.03664 0.03803 1.402 0.16081  MEAN_MW 0.10005 0.08744 0.09071 1.103 0.27005  Conventional models  Component models:  df logLik AICc Delta Weight  123 5 -23.64 59.16 0.00 0.59  1234 6 -23.62 61.96 2.80 0.14  1235 6 -23.63 61.97 2.81 0.14  12 4 -27.26 63.72 4.57 0.06  12345 7 -23.62 64.96 5.81 0.03  125 5 -27.25 66.37 7.21 0.02  124 5 -27.26 66.39 7.23 0.02  1245 6 -27.25 69.20 10.05 0.00  Term codes:  arabkm1z as.factor(CALYEAR) MEAN_MW smeanhb SP_CODE_TOT_B  1 2 3 4 5  Model-averaged coefficients:  Estimate Std. Error Adjusted SE z value Pr(>\|z\|)  (Intercept) 1.799700 0.210346 0.216769 8.302 <2e-16  MEAN_MW -0.133264 0.050193 0.052067 2.559 0.0105  arabkm1z -0.003856 0.080840 0.083839 0.046 0.9633  as.factor(CALYEAR)2003 0.335806 0.161928 0.167860 2.001 0.0454  smeanhb -0.004084 0.027364 0.028406 0.144 0.8857  SP_CODE_TOT_B 0.001785 0.017446 0.018107 0.099 0.9215 |
| --- |
|  |
| \|  \| \| --- \| |
